# Dissociating social reward learning and behavior in alcohol use disorder

**DOI:** 10.1038/s41398-025-03236-3

**Published:** 2025-01-25

**Authors:** Simon Jangard, Björn Lindström, Lotfi Khemiri, Nitya Jayaram-Lindström, Andreas Olsson

**Affiliations:** 1 Centre for Psychiatry Research, Department of Clinical Neuroscience, Karolinska Institutet & Stockholm Health Care Services, Region Stockholm, Stockholm, Sweden; 2 Division of Psychology, Department of Clinical Neuroscience, Karolinska Institutet, Stockholm, Sweden; 3 Department of Medical Epidemiology and Biostatistics, Karolinska Institutet, Stockholm, Sweden

**Keywords:** Human behaviour, Addiction

## Abstract

**Background:**

Alcohol use disorder (AUD) is associated with deficits in social cognition and behavior, but why these deficits are acquired is unknown. We hypothesized that a reduced association between actions and outcomes for others, i.e., social reward learning, would explain prevalent social deficiencies in AUD.

**Methods:**

We conducted one laboratory study (*n* = 234) and one confirmatory online study (*n* = 258), comparing young adults with AUD to age-, gender-, and education-matched healthy controls on a standardized reward learning task. In the task, participants learned to maximize reward for another person and for oneself. To elucidate the potential relation between reward learning and social behavior in AUD, we administered two measures: a dictator game task and a self-report measure. Finally, we applied reinforcement learning models to examine the computational properties of learning.

**Results:**

Social and individual learning, expressed in choice behavior, were comparable in individuals with AUD and healthy controls. Individual differences in learning were not associated with reduced social behavior in AUD. Computational modeling suggested that the learning mechanisms are comparable in AUD and healthy controls and indifferent to whether learning maximizes reward for another person or oneself.

**Conclusions:**

Individuals with AUD demonstrated preserved reward learning abilities that do not vary with social behavior. Together, these results indicate that reward processes may not be relevant for understanding compromised social behavior in AUD.

## Introduction

Compromised social behavior has been suggested as a risk factor in the initiation of alcohol use disorder (AUD) [1–4]. However, it is presently unclear why social behavior is reduced in AUD as compared to the healthy population. A two-fold answer may lie in the reward system of the brain that plays a key role in both learning to adapt behavior in healthy social relationships [5] and for ascribing relatively lower value to non-alcoholic rewards as compared to alcohol in AUD [6]. Here, we examined a reward learning mechanism that contributes to social behavior, and that may be impaired in individuals with AUD. To this end, we applied a computational reinforcement learning (RL) framework to understand the formation of associations between one’s own actions and rewarding outcomes for others: prosocial learning.

To date, most of the research on prosocial behavior in AUD has focused on decision-making or self-report measures [3, 7–9]. For instance, previous studies have found an association between AUD and reduced altruistic decisions [3] (i.e., helping without expectations of self-reward) as well as an increased tendency to reject unfair offers from others [10–12]. Reduced prosocial behavior, such as altruism, has also been demonstrated to be associated with cocaine use disorder [13], as well as alcohol- [14] and cannabis use [15], physical aggression [16], psychopathic personality traits [15–17] and attention-deficit/hyperactivity disorder [18]. Together, these findings of reduced prosocial behavior across different psychiatric conditions highlight the role of similar underlying mechanisms (e.g., reward-related processes), resonating with the Research Domain Criteria (RDoC) initiative emphasizing transdiagnostic social biomarkers in psychiatry [19].

Alterations in social reward processes [20] and reduced pleasure from non-drug rewards [21] have been suggested to underlie social interactive deficits in SUD. For example, a study on social motivation has shown that emotional engagement during joint attention with others is diminished in cocaine use disorder [22], and a tendency to disengage from social and occupational activities has been linked to reduced experience of non-drug rewards in AUD [21, 23]. Moreover, a systematic review of clinical and non-clinical samples found a strong association between AUD symptom severity and an increased preference for alcohol compared to alternative rewards [6]. Despite the relevance of reward processes for understanding social interaction deficits in SUD, no studies have specifically examined how these processes shape prosocial behavior in individuals with SUD. Addressing social forms of reward learning in AUD would provide a detailed understanding of our previous findings on why individuals with this diagnosis demonstrate reduced prosocial behavior [3]. However, if social reward learning remains intact in AUD, this could indicate that prosocial deficits in AUD and related disorders are independent of underlying learning mechanisms.

Reinforcement learning modeling provides a framework for understanding various aspects of the learning process beyond task-based choice behavior (e.g., reward learning rate: the degree to which a change in choice depends on the difference between experienced and expected reward (Sutton and Barto [24]). The modeling framework has uncovered similarities across conventional psychiatric diagnoses and suggested novel treatment targets [25–28], in line with the RDoC Initiative [29, 30]. For instance, in individuals with anxiety or depression, reinforcement learning modeling demonstrates a reduced reward learning rate in both social and non-social contexts [25, 28] whereas for AUD, reward learning rate has only been investigated in non-social contexts where it is reduced in comparison to the healthy population [31, 32]. However, given the role of compromised social cognition for AUD [7, 8], learning rates in a social context could represent a novel treatment target [28].

The present study aimed to investigate social reward learning in young adults with AUD compared to a matched sample of healthy individuals. We conducted two separate experiments using a standardized probabilistic reinforcement learning task designed to separate rewards for another person (i.e., prosocial learning) from rewards for self (i.e., individual learning) and rewards for no one (i.e., control condition), respectively [33]. Additionally, we investigated the relation between social reward learning and behavior using a standardized dictator game task and a self-report measure. We tested three main hypotheses: Firstly, we hypothesized that young adults with AUD, compared to healthy individuals, would exhibit reduced prosocial learning in terms of choice behavior. Secondly, we hypothesized that reduced prosocial learning in AUD would be associated with reduced prosocial behavior. Thirdly, we hypothesized that reinforcement learning modeling would demonstrate a mechanism of reduced prosocial learning rate in AUD.

## Materials and methods

### Participants

Participants in the study were young adults (aged 18–24) with moderate to severe AUD, and age, gender, and education-matched healthy controls (HC) for comparison. They were recruited as part of two separate studies: (1) A Swedish laboratory study conducted at the Karolinska Institutet, comprising 119 individuals with AUD (50% males), and 115 healthy individuals (50% males) who were recruited and tested in person; (2) An American online study comprising 123 individuals with AUD (51% males), and 135 healthy individuals (40% males) via the Prolific Research Platform (www.prolific.co). Details regarding power estimation, recruitment, and selection process are provided in Supplementary Methods.

The AUD group fulfilled a minimum of four DSM-5 criteria for AUD (corresponding to a moderate or severe AUD) and had to be abstinent on test day and the day before testing. Exclusion criteria for both groups included substance use disorder, neurodevelopmental disorder (e.g., ADHD), and severe psychiatric disorder requiring treatment. For the laboratory study, assessment of the clinical criteria using the Mini International Neuropsychiatric Interview [34] was done by a licensed psychologist, while for the online study, assessments were self-rated by the participants [35]. In the laboratory study, verbal and non-verbal IQ scores were assessed using the WAIS-IV vocabulary and working memory/digit span subtests, respectively [36]. Full inclusion and exclusion criteria and assessment information are provided in Supplementary Methods.

All participants were given a compensation of three movie vouchers (lab study) or $16 (online study) for their participation. They were also promised a bonus based on their performance during the tasks, which resulted in the same bonus for all: 1 additional movie voucher (lab study) or $4 (online study). All participants provided their informed consent, and the procedures were in accordance with the Declaration of Helsinki. The laboratory study was approved by the Swedish Ethical Review Board (Dnr: 2019-05123). No ethical approval was required for the online study as the Swedish Act concerning the Ethical Review of Research Involving Humans (2003:460) states that approval is needed only when personal data is handled. A pre-registration of the study, including a general plan for analysis, can be found on the webpage for the Center for Open Science: https://osf.io/25d8c/.

### Behavioral tasks

#### Prosocial learning

**Fig. 1:**
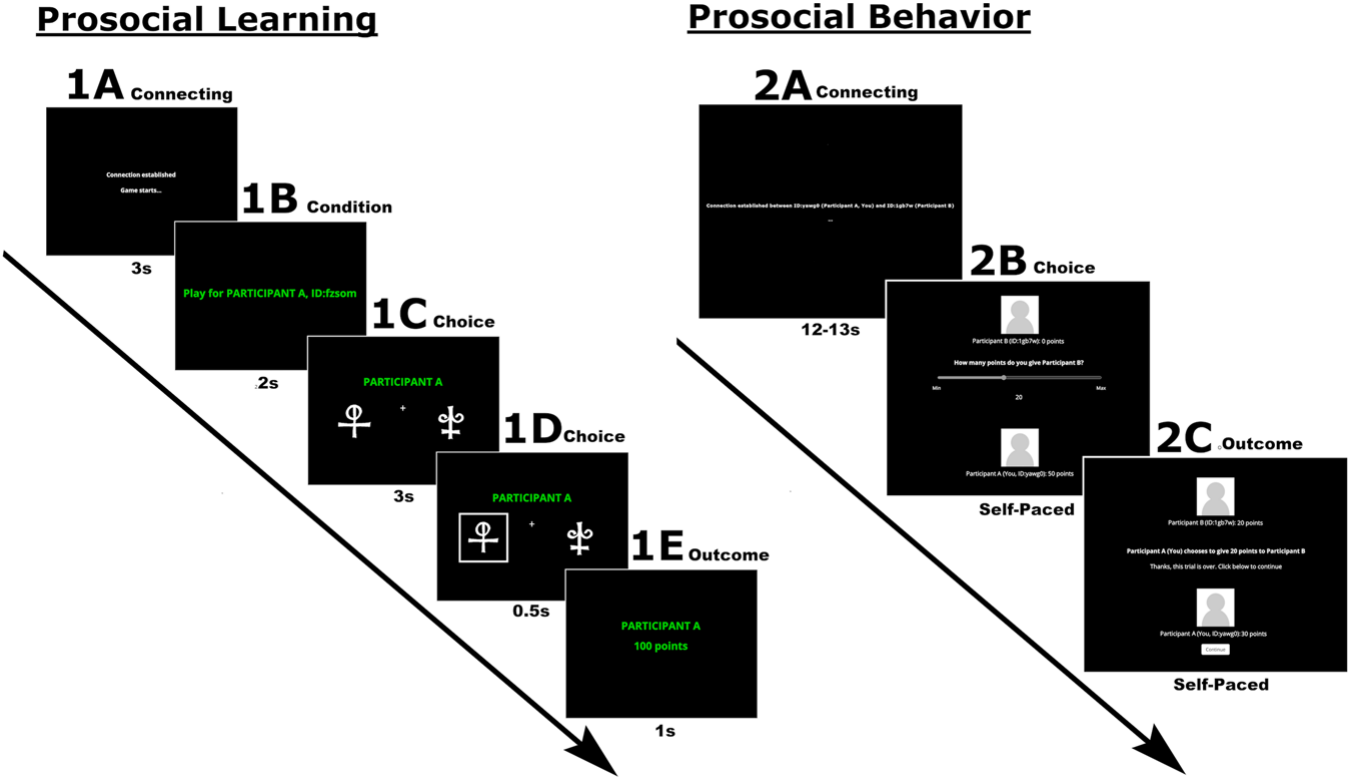
Reward Reinforcement Learning Task (1) assessing prosocial learning in 48 trials, and Dictator Game Task (2) assessing prosocial behavior in 2 trials. Experimental timelines showing: **1A** connection established to another participant, **1B** start of prosocial condition, **1C**, **D** playing participant chooses stimuli for the other participant, **1E** outcome, and **2A** connection established to another participant, **2B** playing participant chooses how many points to give to the other participant, **2C** Outcome.

Participants’ prosocial learning was estimated using a probabilistic reinforcement task which is a slightly modified version of the task used in [33], see Fig. 1A, and Supplementary Methods. For validation of the social reward learning task, see Supplementary Results. The task was implemented in the Presentation® software (Neurobehavioral Systems, Inc., Berkely, CA, http://www.neurobs.com) (lab study), and jsPsych [37] (online study). Participants were informed that they would play with the same and allegedly real interaction partner over an online network during the task (see Supplementary Methods and Supplementary Table S5). The participants were instructed to choose one of two symbols to be rewarded with economically incentivized points over a set of trials. Participants were instructed that the interaction partner would receive any points they won. Apart from prosocial learning, which is the main interest of this study, the participant performed the task in two additional learning conditions: self, where the participant would receive any points, and no one, where no person would receive any points (see Supplementary Methods for full instructions given to participants). Each condition consisted of 48 trials subdivided into three different blocks, resulting in 144 trials overall (see Supplementary Methods for trial structure).

#### Prosocial behavior

Prosocial behavior in terms of altruism was assessed using two separate measures: a dictator game task, and a self-report measure. The dictator game task consisted of 2 trials and was implemented in PsychoPy (lab study), and jsPsych (online study) [37, 38]. Participants were informed that they would play online with a different and allegedly real interaction partner for each trial of the task (i.e., a “one-shot” task [39], see Supplementary Methods in [3] for instructions and details). The participant decided on the distribution of 50 points for oneself and/or another participant (see Supplementary Methods for illustration). Prosocial behavior was operationalized as the number of points transferred to the other participants. The results from this task on our study samples have previously been reported in [3]. As a second confirmatory measure of prosocial behavior, the altruism-subscale of a self-report measure assessing situation-dependent prosocial behavior: the Prosocial Tendencies Measure, for scale items, see Supplementary Methods [40].

### Self-report measures

Alcohol and substance use were assessed with the Alcohol Use Disorder Identification Test (AUDIT, [41]) and Drug Use Disorder Identification Test (DUDIT, [42]) respectively. Abstinence duration and alcohol use in the past 30 days were assessed using the timeline follow-back method [43] while alcohol craving was assessed using a single-item visual analog scale (ranging from 0 to 100, asking “How much craving for alcohol do you experience right now?”) [44]. Duration of harmful drinking was assessed using the single item “For how long (years; months) would you consider that you have been drinking too much?”. Additional psychiatric symptom load was assessed with the Depression Anxiety Stress Scales-21 [45], the Adult ADHD Self-Report Scale-6 (Kessler et al., [46]), and the Autism Spectrum Quotient-10 [47]. Social network size was assessed using a single item derived from the Social Network Index [48]: “How many close friends do you have? (meaning people that you feel at ease with, can talk to about private matters, and can call on for help)”.

### Statistical analysis

All models were estimated in R (version 4.04) using the lme4-package (version 1.1-26) [49] and the GFD-package [50] see Supplementary Methods. Unless otherwise noted, we used a maximal random effect structure in our models as this has been recommended for best generalization [51]. Group differences were investigated by conducting separate multilevel logistic regression analyses for the Swedish lab sample and the confirmatory US online sample since our main interest was to identify group differences that were replicated in both samples. We applied multilevel logistic regression using the glmer function from the lme4 package, specifying a binomial error distribution with a logit link function.

Our first hypothesis stated that AUD would exhibit reduced prosocial learning in terms of choice behavior. We tested this by using high probability choice (i.e., choosing the symbol that was associated with reward in 75% of the trials) as our binary outcome variable, which we modeled as a function of group (AUD vs. Control, fixed effect), condition (Self vs. Prosocial vs. No one, fixed effect), and the interaction between the two (see M1). Additionally, we investigated whether there was an effect of group on the speed of learning (see Supplementary Methods). Variation across trials and conditions for each participant was accounted for with a random intercept. Variation across participants in the effect of Condition was modeled with a random slope. We present the models using the *lme4* syntax [49]:

$${\rm{M}}1:{HighProbChoice} \sim {Group}+{Condition}+{Group}:{Condition}+(1+{Condition}|{Participant})$$

Our second hypothesis stated that prosocial learning would be associated with prosocial behavior in AUD. We tested this by expanding M1 by separately adding task-based and self-reported prosocial behavior as a fixed effect and as an interaction term together with group and condition:

$${\rm{M}}2:{HighProbChoice} \sim {Group}+{Condition}+{ProsocialBehavior}+{Group}:{Condition}+{Group}:{ProsocialBehavior}+{Condition}:{ProsocialBehavior}+{Group}:{Condition}:{ProsocialBehavior}+(1+{Condition|Participant})$$

### Computational modeling

Our third hypothesis stated that AUD would demonstrate a reduced computational mechanism in terms of prosocial learning rate. We tested this by modeling learning for each participant during the task with a standard reinforcement learning algorithm [24]. The basic model has two free parameters that correspond to different mechanisms, which can be estimated from choice data: (1) the learning rate parameter α (i.e., alpha) which is the target of the third hypothesis, and determines the influence that prediction errors—the difference between experienced and expected rewards—has on future reward expectation, and (2) the temperature parameter β (i.e., beta) which represents the noisiness or randomness of decisions. For more details, see Supplementary Methods.

We fitted the data by comparing three models with different complexities. First, following [33], we used a six-parameter model including separate parameters for learning rate and temperature for each of the three conditions Prosocial, Self, and No one. In addition to the full, six-parameter model, we also fitted the behavioral data using two simpler models. First, we considered a model where β was the same across the three conditions (leaving 4 free parameters). Second, following [33], we examined a model where both α and β were the same across conditions (leaving 2 free parameters).

After estimating model parameters for each participant under each model, we used a model selection approach based on the Bayesian Information Criterion (BIC) that balances the fit of the model to individual subject’s data against the complexity of that model. Finally, we report the distribution of the fit parameter values for all examined models [52].

## Results

### Demographics

The demographics and clinical characteristics of the AUD and HC individuals are presented in Table 1. The two groups did not differ regarding sociodemographic variables of gender. With regard to age and education, in the online study, individuals with AUD were slightly older and more educated, but these differences did not affect the main results (Supplementary Tables S6–S7A). In addition, socioeconomic status in terms of income did not affect the main results (Supplementary Table S7B). Finally, the number of close friends did not affect the main results (Supplementary Table S8). As expected, the AUD groups in both samples reported higher levels of drinking, depressive, anxiety, stress, and ADHD symptoms compared to HC. Alcohol had been the main drug of choice during the past year, as confirmed by the low DUDIT scores in both samples [53]. The lab and online study samples were similar in terms of the mean number of fulfilled AUD criteria as well as the self-reported AUDIT score (Table 1).

**Table 1:** Demographics and clinical characteristics.

	Lab study			Online study		
	Alcohol use disorder (*n* = 119)	Healthy control (*n* = 115)		Alcohol use disorder (*n* = 123)	Healthy control (*n* = 135)	
Characteristics			*p*^a^			*p*^a^
Age, Mean (SD)	21.4 (2.0)	21.0 (2.0)	0.111	21.8 (1.7)	20.8 (2.1)	<0.001
Gender, Male, No. (%)	59 (49.6)	58 (50.4)	1.000	63 (51.2)	54 (40.0)	0.086
Socioeconomic Status, Education						
Undergraduate Degree or Higher, No. (%)	42 (35.3)	29 (25.2)	0.124	86 (69.9)	59 (43.7)	<0.001
Socioeconomic Status, Income^b^			0.667			0.074
Don’t Meet Basic Expenses, No. (%)	4 (4.5)	2 (1.7)		8 (6.5)	12 (8.9)	
Just Meet Basic Expenses, No. (%)	8 (9.1)	9 (7.8)		36 (29.3)	23 (17.0)	
Meet Needs with a Little Left, No. (%)	48 (54.5)	64 (55.7)		48 (39.0)	52 (38.5)	
Live Comfortably, No. (%)	28 (31.8)	40 (34.8)		31 (25.2)	48 (35.6)	
Close friends, Mean (SD)	5.9 (1.5)	5.0 (1.8)	<0.001	3.6 (1.8)	3.4 (1.9)	0.458
Non-verbal IQ Score, Mean (SD)	26.3 (4.4)	26.0 (4.0)	0.655	NA	NA	
Verbal IQ Score, Mean (SD)	21.7 (7.6)	21.5 (7.3)	0.884	NA	NA	
Prosocial Behavior						
Dictator Game Task	18.0 (9.9)	21.1 (8.6)	0.013	15.1 (10.3)	19.3 (11.0)	0.002
Altruism Self-Report	20.9 (3.4)	22.4 (2.5)	<0.001	19.6 (4.1)	20.9 (3.8)	0.005
Psychiatric Attributes and Substance Use	Mean (SD)	Mean (SD)	*p*^a^	Mean (SD)	Mean (SD)	*p*^a^
Adult ADHD Self-Report Scale-6	4.5 (2.1)	3.0 (1.8)	<0.001	2.1 (1.4)	1.5 (1.4)	<0.001
Autism Spectrum Quotient-10	2.8 (1.6)	3.0 (1.8)	0.353	3.2 (1.8)	3.6 (2.0)	0.105
Depression Anxiety Stress Scales-21						
Anxiety	3.5 (3.3)	1.2 (1.6)	<0.001	4.7 (3.8)	2.1 (2.6)	<0.001
Depression	6.6 (4.4)	2.7 (2.4)	<0.001	7.8 (5.3)	4.4 (4.3)	<0.001
Stress	6.7 (4.0)	3.6 (3.0)	<0.001	7.1 (4.5)	3.9 (4.1)	<0.001
Alcohol Use Disorder Identification Test	17.3 (4.5)	1.7 (1.8)	<0.001	17.9 (6.2)	1.0 (1.4)	<0.001
Alcohol Use Disorder Criterias, No.	6.2 (1.8)	NA^c^		6.5 (2.1)	0.1 (0.3)	<0.001
Drinking Too Much, No. Months	30.0 (26.7)	1.1 (3.9)	<0.001	27.7 (25.3)	0.8 (6.7)	<0.001
Alcohol Binges, Past 6-months	23.0 (19.0)	NA^d^		18.9 (21.7)	0.1 (0.3)	<0.001
Alcohol Consumption, Past 30-days, No.	51.8 (25.5)	1.4 (3.0)	<0.001	65.6 (39.9)	2.3 (4.6)	<0.001
Alcohol Abstinence, No. Days	3.4 (2.3)	23.7 (9.8)	<0.001	2.9 (2.7)	13.9 (6.0)	<0.001
Alcohol Craving at Time of Testing	18.6 (18.6)	1.2 (7.4)	<0.001	45.3 (26.8)	2.1 (6.9)	<0.001
Drug Use Disorder Identification Test	3.7 (4.5)	0.2 (0.7)	<0.001	1.7 (2.2)^e^	0.3 (0.9)^e^	<0.001

^a^Analysis of variance for continuous variables, or Log-Odds for categorical variables.

^b^Socioeconomic status was assessed using a measure of financial situation [64].

^c^Max 1 criterion for alcohol use disorder required for participation.

^d^Max 1 binge episode required for participation.

^e^The online study used the short-form version of the Drug Use Disorder Identification Test.

### Prosocial learning

**Fig. 2:**
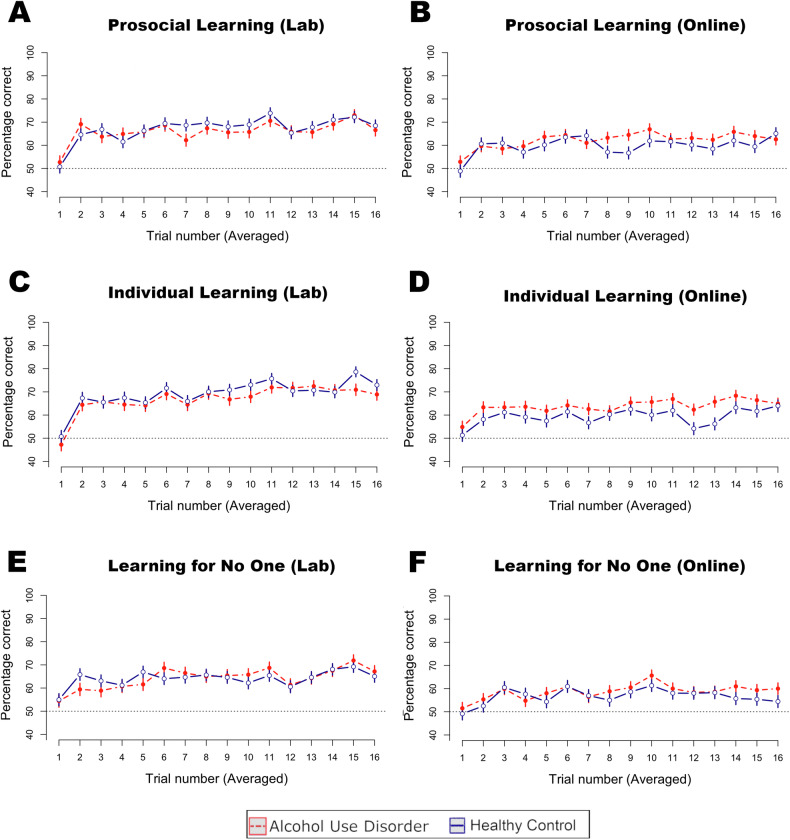
Learning curves across conditions. Learning curves for the Lab **A**, **C**, **E** and Online sample **B**, **D**, **F** in the prosocial, individual, and control conditions show no group difference between alcohol use disorder and healthy control individuals. Trials are averaged over the three blocks (48 trials total per condition, 16 trials per block). The dotted horizontal line shows the chance level.

In testing our first hypothesis, the results indicated no evidence for an effect of AUD on prosocial learning, self-relevant learning, or learning for no one across samples in terms of choice tendency to pick the high-reward stimuli in the separate conditions (Lab study: *χ²* = 0.82, *p* = 0.665; Online study: *χ²* = 3.03, *p* = 0.220). In addition, there was no effect of AUD on the main effect of choice independent of condition (Lab study: *χ²* = 0.08, *p* = 0.781; Online study: *χ²* = 2.49, *p* = 0.114). Next, we added the trial number to the model (Fig. 2). Again, there was no interactive effect of AUD and trial number on choice across the separate conditions (Lab study: *χ²* = 4.20, *p* = 0.122; Online study: *χ²* = 4.43, *p* = 0.109, Supplementary Table 2B).

We verified the robustness of these results with a set of additional analyses. First, the ability to learn across both groups was validated by showing an average performance above the chance level (Supplementary Table S3). Second, non-learners were similar across groups and removed in an additional set of analyses which did not affect the main results (Supplementary Table S4). Third, given that there may be group differences in practice effects from doing the same condition three times, the first block only from each of the conditions was selected for analysis (see Supplementary Table S8). Indeed, there was no group difference across blocks. Fourth, the difference score in trial-by-trial choice behavior was assessed by contrasting the prosocial and self-condition, which did not differ by group (see Supplementary Tables S9). Finally, the group variable in the main models was exchanged for harmful alcohol use past year: AUDIT [41], as well as the number of fulfilled DSM-5 criteria for AUD (including a subset of the social criteria for AUD, Supplementary Tables S11). Although the online study demonstrates some support for a general reduction in learning when using alternative measures of AUD (see Supplementary Tables S11, S12A, B), these results are close to the threshold of statistical significance and did not replicate in the lab study.

Next, considering the potential influence of individual heterogeneity in drinking trajectory and psychiatric symptom load, we investigated the additional influence of alcohol craving, days of alcohol abstinence, alcohol use past 30 days, duration of harmful drinking as well as symptoms of anxiety, depression, stress, ADHD, and Autism. The results showed no consistent influence of drinking trajectory or psychiatric symptoms in AUD on prosocial or self-relevant learning (see Supplementary Tables S12–S20). However, some inconsistent effects were found within each group such that higher symptom severity was associated with increased self-relevant learning for the AUD group in the lab study (see e.g., Supplementary Tables S15B, S16B and S17B). Despite these inconsistencies, the main analyses indicate that both prosocial and self-relevant learning is preserved in AUD despite possible heterogeneity in psychiatric symptom profile.

As a final exploratory analysis, we assessed the impact of reward history on current choice. This is based on the observation that reinforcement learning in substance use disorder has demonstrated recency bias (i.e., repeating the most recent prior responses regardless of past success) [54, 55]. In summary, these results showed no group differences in recency bias (Supplementary Table S17).

### The relationship between prosocial learning and behavior

In the context of the second hypothesis, prosocial behavior is reduced across both the lab and the online study samples (Table 1; Jangard et al. [3]). In testing our second hypothesis on the effect of prosocial learning on behavior, we found no such effect for the task-based measure (Lab study: *χ²* = 0.07, *p* = 0.963; Online study: *χ²* = 0.16, *p* = 0.924) or the self-report measure (Lab study: *χ²* = 0.64, *p* = 0.728; Online study: *χ²* = 3.38, *p* = 0.185). Neither were there any effects of self-relevant learning or learning for no one in relation to the task-based or the self-report measures of prosocial behavior (Supplementary Tables 22–23).

### Computational modeling of prosocial learning

**Fig. 3:**
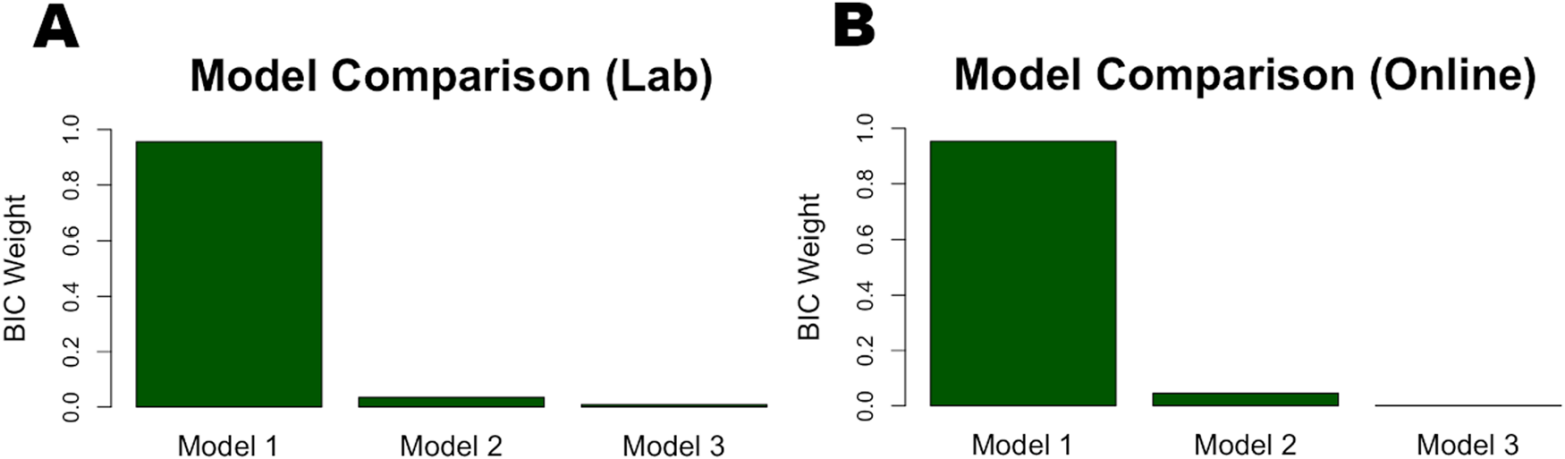
Model comparison using BIC weights. Model selection using BIC weights (i.e., the conditional probability) for the Lab **A** and the Online sample **B** showing Model 1 (i.e., the two-parameter model) to best fit the data. BIC = Bayesian Information Criterion.

To test the third hypothesis on reduced prosocial learning rate (α) in AUD, we applied three reinforcement learning models to the data with increasing complexity. Average model fit, defined in terms of the BIC determined that the smaller two-parameter model where both α and β (i.e., temperature) were the same across the prosocial, self, and no one conditions, provided the best fit to the data (Lab study: ΔBIC > 22.60; Online study: ΔBIC > 22.08). Similarly, BIC model selection using BIC weights [56] demonstrated that the two-parameter model had the highest conditional probability (Fig. 3, Supplementary Table 27). For details of the model fitting, see Supplementary Tables 25–27. Due to the two-parameter model providing the best fit, we focused on it in subsequent analyses.

**Fig. 4:**
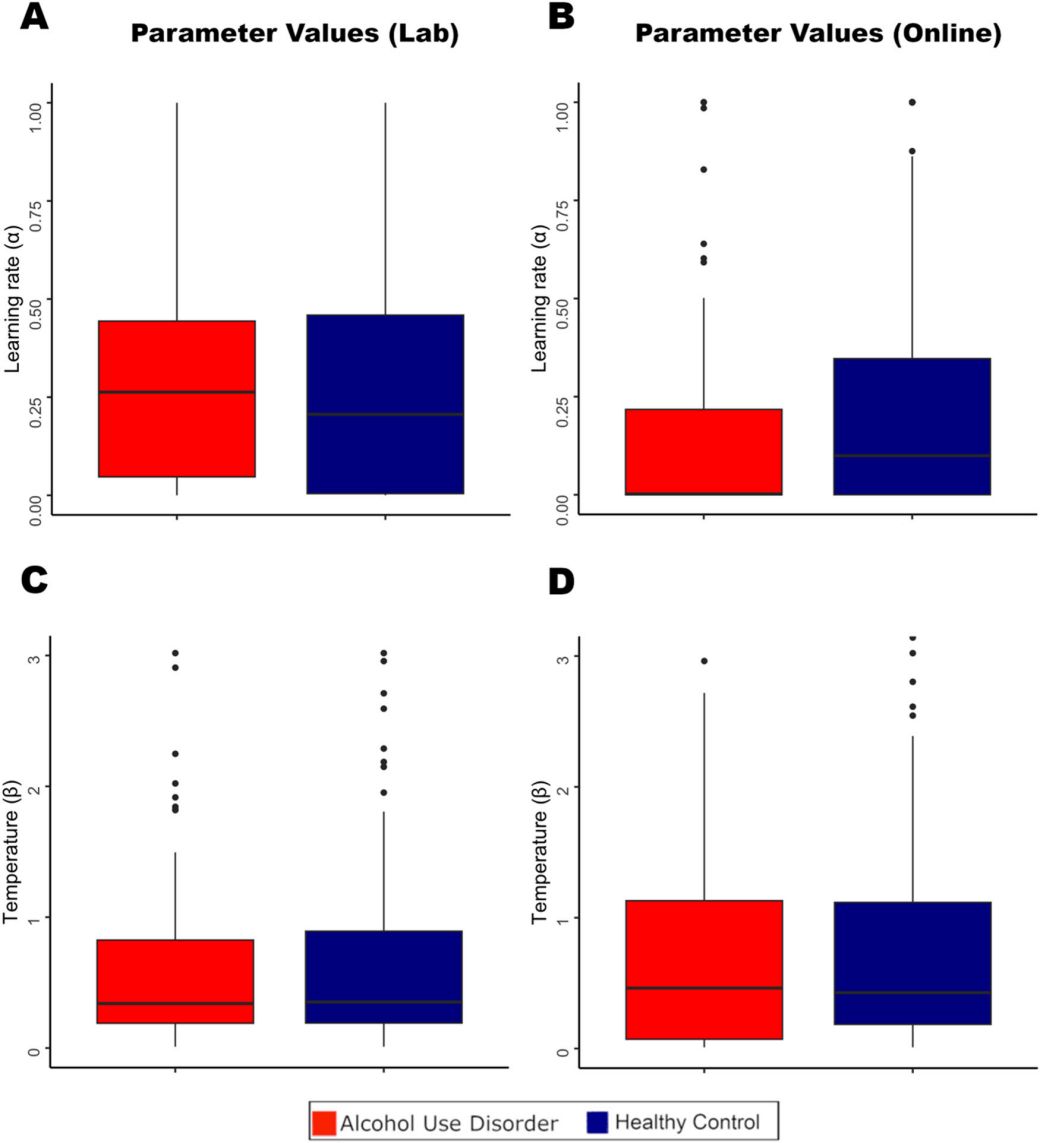
Estimated parameter values for the winning model. Estimated parameter values for the winning model for the lab study **A** and **C** and online study **B** and **D**, with two free parameters applied to individual subject data. There were no significant group differences for estimated values for learning rate (alpha) or temperature (beta) across the three conditions.

Since the estimated parameters derived from each subject in the AUD and HC groups were not normally distributed (Supplementary Fig. S15), a non-parametric Wilcoxon rank sum test was used in the following analyses. The model fit the data from the AUD and HC groups equally well, as indicated by no significant differences between groups in BIC (Lab study: *p* = 0.905; Online study: *p* = 0.301). In contrast to the third hypothesis, there was no consistent effect of AUD on the learning rate parameter (Lab study: *p* = 0.325; Online Study: *p* = 0.060), Supplementary Table S28 and Fig. 4. Additionally, there was no effect of AUD on the temperature parameter (Lab study: *p* = 0.823; Online Study: *p* = 0.407).

## Discussion

In the present study, we used a learning task, combined with computational modeling, to better understand possible processes that could differentiate social learning in young adults with AUD in comparison with healthy individuals. To increase the reliability of our results, we applied the same task in two separate samples; one laboratory sample and one confirmatory online sample. The study yielded three major findings. First, using a standardized social reward learning task, choice behavior, including learning curves did not differ between the two groups. Second, performance on the learning task was not related to reduced prosocial behavior in the AUD compared to the HC group. Third, applying computational modeling, we found that the estimated values for the learning rate did not differ between the task conditions and were comparable between AUD and HC. To our knowledge, this is the first study to investigate social reward learning in AUD and demonstrate intact functioning in these processes in comparison to healthy individuals.

Previous research on individual learning tasks with rewarding outcomes for oneself has yielded mixed findings, with some demonstrating that individuals with AUD show less correct choice behaviors [31] whereas others show preserved choice behavior in comparison to an HC group [57, 58]. We extend these findings by showing for the first time that learning is preserved in AUD independent of whether a choice with rewarding outcomes is for someone else or oneself.

Individual differences in social behavior have been suggested to depend on an underlying learning process [26]. If such a relationship exists in AUD, we would expect an association between prosocial learning and behavior. However, our results were counter to this conjecture, indicating that reward learning and social behavior are separate processes in AUD and related psychiatric conditions. One interpretation of this discrepancy may be that time-dependent learning of associating reward with others or oneself relies on executive processes more robustly to the influence of alcohol compared to e.g., single decisions without feedback where you rather have to trust your own judgment [10].

A mechanism such as learning rate is possible to assess using reinforcement learning modeling, and previous findings on individual learning demonstrate reduced learning rate for AUD [31, 32] and other SUD conditions [55]. Our results extend and contrast previous findings by showing a preserved learning rate both during social and individual learning [59, 60]. However, previous studies differ from the current one by being applied in an inpatient setting including participants of older age which might explain our discrepant findings in the current study [31, 32]. Moreover, the participants’ choices in our study were best characterized by a two-parameter model with the same learning rate for prosocial and self-relevant choices, suggesting that the learning mechanism is similar across social and self-relevant contexts in AUD (cf. [33, 61]). In sum, the result of the present study extends previous findings by showing that young adults with AUD compared to HC groups show no detectable differences in learning rates when associating rewards with oneself and others.

The present study has limitations in need of discussion. First, the study participants in our sample were young adults ranging from 18–24 years of age. Previous studies have demonstrated increased deficits in executive functions in AUD in older age groups [7] and our results do not examine possible social learning deficits in relation to AUD at a later stage. Second, heterogeneity between the lab and the online samples may limit the generalizability of the findings, e.g., some exploratory findings on high symptom levels in AUD in one of the samples are close to the threshold of statistical significance. A third limitation is that our social reward learning task is a model-free learning task (i.e., learning of direct action-outcome contingencies), which does not account for model-based learning mechanisms of relevance to AUD (i.e., learning of sequential action-outcome contingencies requiring a mental model of the task environment) [26, 62–64]. In other words, the present results could not exclude the possibility of social learning deficits in AUD in richer and more complex learning environments. Finally, the design of the learning task in the current study does not include environmental volatility (i.e., unpredictable changes in the learning environment), which has been demonstrated to be relevant for identifying deficits in non-social learning for AUD [32] and social learning for other psychiatric patient groups [59, 60].

In conclusion, to the best of our knowledge, the current study is the first to test social reward learning in AUD. We show that AUD exhibits preserved prosocial learning verified in two separate samples by using both choice data and computational modeling. Future directions include the need to measure volatility [32] as well as model-based learning processes underlying social learning [62]. Moreover, the processes underlying social deficits in AUD may also be addressed using task-based behavioral measures based on, for example, social value orientation [63]. Taken together, the study shows that young adults with AUD retain their ability to learn from rewards when benefiting others or themselves, which suggests that alternative processes may underlie compromised social behavior in AUD.

## Supplementary information

Supplementary Information

## Data Availability

The code and data used for the current study are available from corresponding author Simon Jangard upon reasonable request.
