# Supplementary material for: Dissociating social reward learning and behavior in alcohol use disorder
Source: Transl Psychiatry. 2025 Jan 25;15:30. doi: 10.1038/s41398-025-03236-3 (PMC11762780; doi:10.1038/s41398-025-03236-3)
Supplement: Supplementary file 1 — Supplementary Information [file 41398_2025_3236_MOESM1_ESM.docx]

**Dissociating Social Reward Learning and Behavior in Alcohol Use Disorder**

**Supplementary Information**

Table of Contents

[Supplementary Methods 3](#_Toc178952325)

[Participants 3](#_Toc178952326)

[Power Estimation 3](#_Toc178952327)

[Criteria for Participation 3](#_Toc178952328)

[Recruitment Process and Data Collection 4](#_Toc178952329)

[Prosocial Learning task 4](#_Toc178952330)

[Bonus Compensation 4](#_Toc178952331)

[General Instructions 4](#_Toc178952332)

[Task-Specific Instructions 6](#_Toc178952333)

[Task Illustrations 8](#_Toc178952334)

[Trial Structure 9](#_Toc178952335)

[Prosocial Behavior Task 9](#_Toc178952336)

[Funnel Debriefing 9](#_Toc178952337)

[Statistical analysis 11](#_Toc178952338)

[Exclusion of Null-Responses 11](#_Toc178952339)

[The Effect of Group on the Speed of Learning 11](#_Toc178952340)

[Reinforcement Learning Modeling 12](#_Toc178952341)

[Supplementary Results 13](#_Toc178952342)

[Validating the Prosocial Learning Task 13](#_Toc178952343)

[Group Differences in Prosocial Learning 14](#_Toc178952344)

[Models for Validating the Prosocial Learning Results 16](#_Toc178952345)

[Controlling for Doubt in Cover Story 18](#_Toc178952346)

[Controlling for Differences in Age 19](#_Toc178952347)

[Controlling for Differences in Socioeconomics in terms of Education 20](#_Toc178952348)

[Controlling for Differences in Socioeconomics in terms of Income 21](#_Toc178952349)

[Controlling for Differences in Number of Close Friends 22](#_Toc178952350)

[Controlling for Practice Effects 23](#_Toc178952351)

[Difference Score between Prosocial and Self-Condition 24](#_Toc178952352)

[Exchanging the Group Variable for Self-Reported Alcohol Use Past Year: AUDIT 25](#_Toc178952353)

[Exchanging the Group Variable for Fulfilled AUD Criteria 26](#_Toc178952354)

[Exchanging the Group Variable for Fulfilled ‘Social’ AUD Criteria 27](#_Toc178952355)

[Models Complementing the Group Variable with Additional Psychiatric Processes 28](#_Toc178952356)

[Alcohol Craving 29](#_Toc178952357)

[Days of Abstinence 30](#_Toc178952358)

[Alcohol Use in the Past 30 days 31](#_Toc178952359)

[Harmful Drinking History 35](#_Toc178952360)

[Anxiety Symptoms 37](#_Toc178952361)

[Depression Symptoms 39](#_Toc178952362)

[Stress Symptoms 40](#_Toc178952363)

[ADHD Symptoms 41](#_Toc178952364)

[Autism Symptoms 42](#_Toc178952365)

[Recency Bias 43](#_Toc178952366)

[Associations between Prosocial Learning and Prosocial Behavior 44](#_Toc178952367)

[Prosocial Behavior Using a Dictator Game Task 45](#_Toc178952368)

[Prosocial Behavior Using a Self-Report Measure 46](#_Toc178952369)

[Computational Modeling of Prosocial Learning 47](#_Toc178952370)

[Supplementary References 49](#_Toc178952371)

# Supplementary Methods

## Participants

### Power Estimation

We aimed for a sample size of 120 per group for the laboratory study, and it was based on a college sample showing an estimated effect size of 0.12 of self-reported prosocial behavior on substance abuse (1). We used G*Power 3.1, 95% power, and a two-tailed t-test of two independent means as a basis for our estimation. For the online study, we aimed for 150 participants per group.

### Criteria for Participation

Inclusion criteria for both groups were being between 18 and 24 years of age, and abstinent from drinking on test day, and the day before testing. Additional inclusion criteria for the AUD group were consumption of at least 5 drinks within 2 hours on at least 2 occasions the last 6 months (in accordance with US binge-drinking criteria, of particular importance for this age group), and fulfil a minimum 4 DSM-5 criteria of AUD. The HC group, on the other hand, could not fulfil the criteria for AUD and needed to report a maximum of 5 drinks within 2 hours on 1 occasion in the last 6 months.

Exclusion criteria for both groups were fulfilling criteria for any other substance use disorder in the past 12 months (apart from nicotine); any other illicit drug use past 1 month (on more than a single occasion); initiation of pharmacological treatment for psychiatric disorders past 3 months; regular psychological treatment in the past 3 months; severe psychiatric disorder (e.g., severe depression; bipolar-, personality, or psychotic disorder) past 12 months; extended psychiatric care before alcohol use onset; diagnosis of neurodevelopmental disorder, including ADHD, Autism and Tourette’s; severe medical conditions requiring medication (e.g., epilepsy); neurological conditions (e.g., multiple sclerosis); history of head trauma (i.e., loss of consciousness for longer than 2 minutes).

All study participants were screened for inclusion-, and exclusion criteria and the DSM-5 diagnostics were assessed using the Mini International Neuropsychiatric Interview (3). For the lab study, a clinical psychologist or a psychologist in training performed the pre-assessment on the telephone, and a confirmatory in-person assessment. For the online study, the assessment was based on a self-report screening questionnaire before the full study, and the additional requirement for the AUD/HC group to fulfill/not fulfill the criteria of hazardous alcohol use on the Alcohol Use Disorder Identification Test (AUDIT) (4). The lab study did in addition assess verbal IQ using the WAIS-IV vocabulary subtest, and working memory using the digit-span subtest (5)

### Recruitment Process and Data Collection

Participants were either recruited through advertisements on addiction clinics, social media, and university campuses in the Stockholm area (Lab study), or on the Prolific Research Platform targeting American participants (Online study). The advertisements were phrased based on “having second thoughts as of whether drinking too much” (AUD-group), and “being healthy with low alcohol consumption (HC-group).

For the lab study, data were collected between January 2020 and November 2020 (with a 5-month stop from April to August because of the COVID-19 pandemic). For the online study, data were collected in June 2020.

549 (322 AUD) participants (lab study) and 921 (582 AUD) participants (online study) were screened for participation, out of which 259 (137 AUD) participants (lab study) and 300 (150 AUD) participants (online study) were admitted for participation in the study. 26 participants had to be excluded afterward in the lab study due to technical difficulties during testing or due to information omitted in the telephone interviews but revealed in the in-person clinical assessment (e.g., not fulfilling diagnostic criteria for moderate AUD; previous diagnosis of ADHD, or personality disorders). 51 participants were excluded afterward from the online study due to not fulfilling the criteria of hazardous/non-hazardous [AUD/HC] alcohol use on the Alcohol Use Disorder Identification Test or due to technical difficulties with data collection. Thus, the final sample for statistical analyses consisted of 233 participants in the lab study and 249 participants in the online study (but see Supplementary Methods: Statistical analyses for details on removing null responses and non-learners).

## Prosocial Learning task

### Bonus Compensation

Participants were informed that 10% of all decisions made by themselves and the other participants would be converted into a monetary bonus at the end of the study (for details, see ‘Instructions’ below). However, no exchange rate was revealed before completion of the study, and at the end of the study, all participants received the same bonus in terms of 1 movie voucher (lab study) or $4 (online study).

### General Instructions

Welcome to a study about decision-making!

Before starting the study, we would like you to answer one question on alcohol.

How much craving for alcohol do you experience right now, on a scale from 0-100?

0 = no craving for alcohol at all

100 = strongest possible craving for alcohol; if alcohol was available it would be impossible to resist

The study consists of 5 parts where you will make decisions affecting yourself and others (Part 1-5), and in addition, answer a number of questionnaires (Part 6).

You will be completely anonymous, and will on several occasions be paired with other anonymous participants through our online platform. The other participants will on some occasions play the same game as yourself.

Neither you nor the other anonymous participants will be given any information about each other.

To keep track of each participant on the screen, each participant will be allocated a randomized and anonymous ID number. You will receive your ID number on the next page, which will follow you through the different parts of the study.

You will in the different parts of the game make decisions that affect how many points you and the other participants will receive.

You and the other participants will be paid based on your decisions. Out of all decisions made by you and the other participants, 10% will randomly be selected.

The total amount of points that you and the other participants have received from these selected decisions will be converted to an economic bonus on top of your promised payment for participation.

In other words, the more points you and the other participants earn for yourself and for each other, the larger bonus sum will be paid out to your accounts.

The bonus payment from your decisions will be paid out within a week.

There will be specific instructions for each 5 parts of the game. You will receive these instructions before starting each part. In other words, instructions for Part 2 will be given after you have finished Part 1, etc.

The 5 different parts of the game are independent of each other. In other words, the decisions you make during Part 1 will not affect what happens in Part 2 and so forth.

You will receive a few control questions before the start of each Part to make sure that you understand the game that you are about to participate in.

Control question 1: Will the decisions that you make in Part 1 affect what will happen in Part 2-6?

Control question 2: Are you and the other participants anonymous toward each other?

Control question 3: Will the decisions that you and the other participants make affect the number of points that you and the other participants earn?

Control question 4: Will some of the points you and the other participants earn be converted to bonus payments for you?

### Task-Specific Instructions

Welcome!

This part will take approximately 30 minutes.

This part consists of a Participant A and a Participant B that will be the same persons during the entire first part. You will play the role of Participant B.

*Information about your participation:*

You will play a number of trials where you for each trial will see a pair of symbols and you need to select one of them.

You will receive points for some of your choices which means that the more points you win for yourself the more money you will have in the end.

Some of the symbols will give you points more often than others.

Each symbol has its own meaning, regardless of where it appears on the screen (so left and right are not important) or when it occurs in the task.

You will play in 3 different conditions where you will play for 1) yourself, 2) for the other anonymous participant (Participant A), or 3) for no one.

When you play for yourself, you will receive the money you win.

When you play for the other participant, he/she will receive the money you win. However, the other participant won't know that you are performing a task where you could win extra money for them, and the money you earn for the other participant will be given as an extra bonus for participation.

When you play for no one, neither you nor the other participant will receive any money.

Make your choice by using the left and right arrow keys.

Try to respond as quickly as possible, you have about 2 seconds to make your choice.

You will start by playing a practice session.

During the practice session, you won't keep any money you win.

*[Practice session including 8 trials, always in SELF-condition]*

You have now finished the practice session.

Please sit comfortably. Part 1 will start on the next screen and go on for approximately 25 minutes.

### Task Illustrations


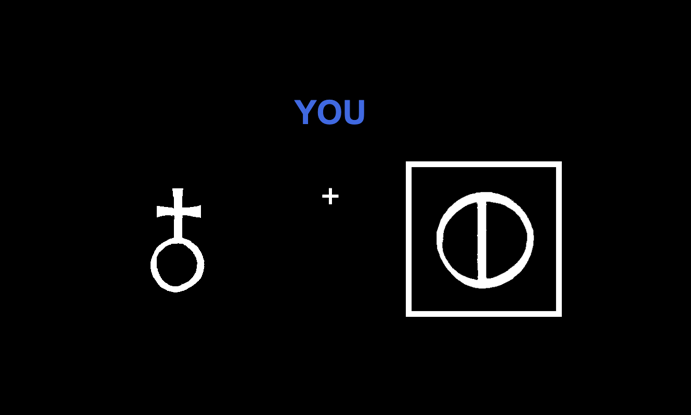


**Supplementary Figure S1.** Prosocial Learning Task, Self-condition. Figure showing: playing participant chooses what symbol to choose with possibility for reward for oneself.


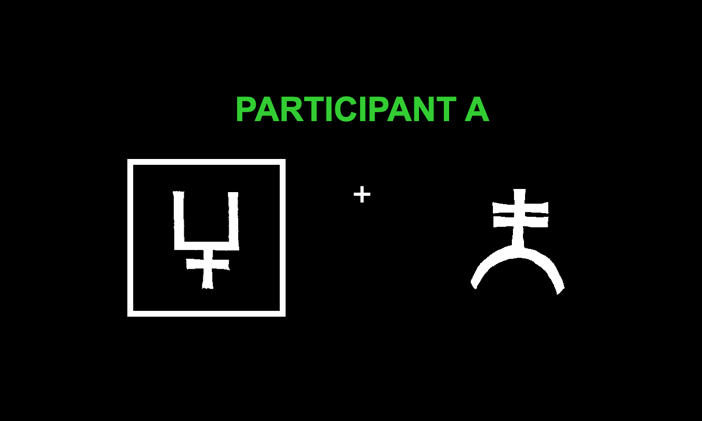


**Supplementary Figure S2.** Prosocial Learning Task, Prosocial-condition. Figure showing: playing participant chooses what symbol to choose with possibility for reward for another participant.


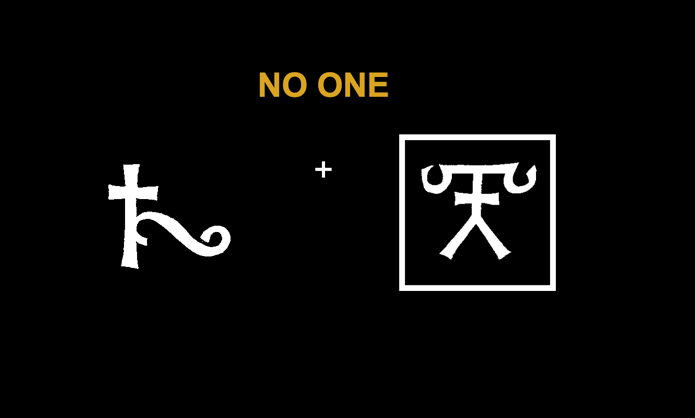


**Supplementary Figure S3.** Prosocial Learning Task, No one-condition. Figure showing: playing participant chooses what symbol to choose with possibility for reward for no one.

### Trial Structure

The trial structure of the behavioral task began with an instruction screen displayed for 2,000 ms, that indicated who would receive the outcome (”Self”, ”Participant B”, or ”No one”). Next, two abstract stimuli, represented by letters from the Agathodaimon font, were presented for 3,000 ms, during which participants had to select one of them. In case no response was made within this timeframe, a red "missed" label appeared on the screen. The chosen option was briefly shown for 300 ms, followed by a delay of 2,500 ms, and finally, the outcome of the selected option was displayed (either a win of 100 points or 0 points). Subsequently, a randomly varied fixation point appeared on the screen for a duration of 2,000 to 4,000 ms before the two symbols were presented again.

To prevent action-based learning, the side of the screen on which the symbols appeared was counterbalanced. Additionally, participants were explicitly instructed that the side of the stimulus had no significance. This ensured that the participant focused on the contingencies between the stimuli regardless of their position on the screen.

There was a total of 144 trials, equally distributed among three conditions: self, prosocial, and no one. These trials were organized into three blocks of 16 trials each, with each block introducing a new pair of symbols to learn. The presentation order of the blocks followed a pseudorandom sequence, ensuring that the same block type was never presented twice in a row. Additionally, the block order was counterbalanced across participants, ensuring that the block order varied across participants.

## Prosocial Behavior Task

Prosocial behavior in terms of behavioral response using a brief dictator game task was previously published (2). More details can be found in the published paper.

Prosocial behavior in terms of response to a self-report measure was assessed using the altruism subscale of the Prosocial Tendencies Measure (Carlo & Randall, 2002). To each of the following items, the participant responded to “How much each statement describes you”, using a 5-point Likert scale (Does not describe me at all; Describes me a little; Somewhat describes me; Describes me well; Describes me greatly):

- I think that one of the best things about helping others is that it makes me look good.

- I believe that donating goods or money works best when it is tax-deductible.

- I think there should be more recognition for the time and energy people spend on charity work.

- One of the best things about doing charity work is that it looks good on my resume.

- I feel that if I help someone, they should help me in the future.

## Funnel Debriefing

Participants were told that they would interact with other randomly assigned study participants. A cover story was presented stating that they would interact with the other subjects via an internet connection. The plausibility of the cover story was controlled for by the following funnel debriefing (interview for lab study, self-report for online study) at the end of the tasks (11). Participants' belief in the cover study had no impact on our results, see Supplementary Results S3.

1. Tell the participant that the study is over. Ask if the participant has any questions. If the questions are about hypotheses or the deceptive elements of the experiment, explain that you will address those specific questions in just a few moments.
2. Ask whether the experiment was clear in its overall purpose and whether all aspects of the procedure made sense. Was there anything that the participant found confusing or unclear? “Were you, at any point, unsure about what we were asking you to do?”
3. We would find it very helpful to hear about any of your personal feelings and reactions to the experiment. Probe about what made the participant feel the way they did.
4. Today’s experiment was designed to help us test some very specific hypotheses about human behavior. Do you have any idea what those hypotheses were? If you had to guess, what would you say were the hypotheses we were testing today? We would like to know as many of your guesses about our hypotheses as you can come up with.
5. Ask whether the participant found any aspect of the procedure odd, upsetting, or disturbing.
6. Did you wonder at any point whether there was more than meets the eye to any of the procedures that we had you complete today? That is, do you think that there might have been any information that I held back from explaining to you about the experiment until now? Ask participant to say more about their suspicions, and to elaborate on their questions about the procedure.
7. Ask how the participant thinks (the suspicions the participant mentioned) affected his or her behavior during the study.
8. The experimenter then fully explained the nature of the deception to the participant and why it was a necessary part of the experiment, namely, to increase the validity of the task.

## Statistical analysis

### Exclusion of Null-Responses

Although we did not pre-register any exclusion criteria for missing responses based on response time, on inspection we identified individual null responses (i.e., failing to respond to a trial within 3 seconds). Given that individuals on these trials likely were not paying attention to the task (e.g., got distracted), we chose to exclude these trials which comprised 427 trials (i.e., 1.3% of all trials) in the lab study and 710 trials (i.e., 2.0% of all trials) in the online study.

### The Effect of Group on the Speed of Learning

In addition to answering our first hypothesis using model M1 in the main manuscript, we also investigated whether there was an effect of group on the speed of learning (see model M1b below). We expanded the model by adding Trial number (1-16) across three blocks, both as a fixed effect and as an interaction term. The fixed effect from trial and the interactive effect of trial and condition were added to the model using a random intercept and a random slope.

Given that learning over trials is not linear but rather follows a curve, we also evaluated a model where the learning variable had a negatively accelerating (i.e., natural logarithmic) effect (4). By comparing the model fit using the Bayesian Information Criteria (BIC) for this alternative model to the standard model with a linear learning variable, we demonstrated that the model with learning as a negative accelerating effect demonstrated the best fit to data (Lab study: ΔBIC > 159; Online study: ΔBIC > 95). Therefore all analyses including the learning variable use this type of transformation.

*Model specification:*

M1b: *HighProbChoice ~ Group + Condition + log(Trial) + Group:Condition + Group:log(Trial) + Group:Condition:log(Trial) + (1 + Condition + log(Trial) + Condition:log(Trial) | Participant)*

## Reinforcement Learning Modeling

The basis of the reinforcement-learning algorithm is the expected value that an action (e.g., choosing Symbol 1 or Symbol 2) will provide a reward on a specific trial.

The reinforcement learning algorithm states that future reward expectations for an action 'a', denoted as Q_t+1_(a), depend on the current expectations Q_t_(a) and the difference between the actual reward received on the current trial t (coded as 1 or 0 for reward or no reward, respectively) and the expected reward for this trial t, Q_t_(a). The extent to which this difference updates the expectation is determined by the learning rate α, which ranges from 0 to 1.

*Q_t+1_(a) = Q_t_(a) + ﻿α ** [*r_t_ – Q_t_(a)*]

The learning rate parameter ﻿α (i.e., alpha) determines the influence that new information (i.e., reward or no reward) has on future reward expectations. A high learning rate means that the participant gives more importance to new information (e.g., by more easily switching the choice of symbol in the face of new information of non-reward). A low learning rate means that that new information affects this expectation to a lesser extent (e.g., by more strongly staying with the choice of symbol despite new information of non-reward).

The probability that a subject chooses action an on trial *t*, given the expected values of the available actions Q_t_(a), is based on the softmax link function:

$$p_{t}\left[ a \right]Q_{t}(a)=\frac{e^{[Qt(a)/\beta]}}{\sum{{a'}^{e}}^{[Qt(a^{'})/\beta]}}$$

The temperature parameter β (i.e., beta) is included in the softmax function and represents the noisiness of decisions— to what degree the participant explores new options or chooses (i.e., exploits) the option with the highest expected value. A high value for β means choices seem random as they are equally likely irrespective of the expected value of each option. A low β leads to choosing the option with the greatest expected value on all trials.

For details on specific formulations of the models for this study, see Supplementary Table 24-26.

# Supplementary Results

## Validating the Prosocial Learning Task

We evaluated the interaction effect of trial number and condition on high probability choice. Given that trial-based learning in the task is not linear over time but rather demonstrates a curvature (e.g., see Supplementary Figure S1), we also evaluated a model where learning had a negative accelerating effect (i.e., natural logarithmic effect). Using BIC we showed that the model specifying learning as a negative accelerating effect demonstrated a better fit to the data compared to the standard model which led us to use this model.

Since we were interested in the main effect of log(trial number) on choice (i.e., the average effect of log(trial number) on choice and not the effect on trial number 0, we chose to evaluate the model results using a type II Wald chi-square tests Anova.

Our result demonstrated the interaction effects of trial number and all conditions respectively in the lab study. However, the online study did not show any interaction effect which indicates similar learning speed across conditions.

*Model specification:*

*glmer(HighProbChoice ~ Condition + log(Trial) + Condition:log(Trial) + (1 + Condition + log(Trial)) | Participant))*

##### **Supplementary Table S1.** Logistic multilevel linear regression model for validating the prosocial learning task including trial number

|  | **Lab Study** | | **Online Study** | |
| --- | --- | --- | --- | --- |
| *Predictor* | *ChiSq* | *p* | *ChiSq* | *P* |
| Condition | 11.39 | **.003** | 19.36 | **<.001** |
| log(Trial) | 113.68 | **<.001** | 47.82 | **<.001** |
| Condition:log(Trial) | 23.01 | **<.001** | 3.06 | .217 |

## Group Differences in Prosocial Learning

Here we tested our main hypotheses regarding group differences between AUD and HC in prosocial learning. Group, Condition, and Group-Condition interaction is nested by ID, due to 16*3(blocks)*3(conditions)=144 trials per participant. Condition is added as a random factor to the model.

*Model specification:*

﻿*glmer(HighProbChoice ~ Group + Condition + Group:Condition + (1 + Condition | Participant))*

| **Supplementary Table S2A.** Logistic multilevel linear regression model for assessing group differences in prosocial learning | | | | |
| --- | --- | --- | --- | --- |
|  | **Lab Study** | | **Online Study** | |
| *Predictor* | *ChiSq* | *p* | *ChiSq* | *p* |
| Group | 0.07 | .781 | 2.49 | .114 |
| Condition | 16.20 | **<.001** | 21.36 | **<.001** |
| Group:Condition | 0.81 | .665 | 3.02 | .220 |

Next, we added learning curves to the model. For transformation of the trial variable see Supplementary Methods.

Since we were interested in the main effect of log(trial number) on choice (i.e., the average effect of log(trial number) on choice and not the effect on trial number 0, we chose to evaluate the model results using a type II Wald chi-square tests Anova.

*Model specification:*

*glmer(HighProbChoice ~ Group + Condition + Log(Trial) + Group:Condition + Group:Log(Trial) + Group:Condition:Log(Trial) + (1 + Condition + Log(Trial) + Condition:Log(Trial) | Participant))*

##### **Supplementary Table S2B.** Logistic multilevel linear regression model for assessing group differences in prosocial learning including trial number

|  | **Lab Study** | | **Online Study** | |
| --- | --- | --- | --- | --- |
| *Predictor* | *ChiSq* | *p* | *ChiSq* | *p* |
| Group | 0.18 | .669 | 1.56 | .211 |
| Condition | 11.63 | **.003** | 19.32 | **<.001** |
| log(Trial) | 113.66 | **<.001** | 48.00 | **<.001** |
| Group:Condition | 0.82 | .662 | 3.08 | .214 |
| Group:log(Trial) | 0.47 | .491 | 1.24 | .265 |
| Condition:log(Trial) | 22.94 | **<.001** | 3.05 | .217 |
| Group:Condition:log(Trial) | 4.43 | .109 | 0.03 | .986 |

## Models for Validating the Prosocial Learning Results

**Ability to Learn in Different Conditions**

Participants in both groups were able to learn to obtain rewards in all three conditions. This was demonstrated through average performance above chance level (>50%, i.e. a mean > .50).

##### **Supplementary Table S3.** Descriptive learning showing average performance above chance level (50%)

|  | **Lab Study** | | **Online Study** | |
| --- | --- | --- | --- | --- |
| *Group (Condition)* | *Mean* | *SD* | *Mean* | *SD* |
| AUD (Prosocial) | 0.67 | 0.47 | 0.60 | 0.49 |
| AUD (Self) | 0.69 | 0.46 | 0.59 | 0.49 |
| AUD (No one) | 0.64 | 0.48 | 0.57 | 0.50 |
| HC (Prosocial) | 0.66 | 0.47 | 0.62 | 0.48 |
| HC (Self) | 0.67 | 0.47 | 0.64 | 0.48 |
| HC (No one) | 0.64 | 0.48 | 0.59 | 0.49 |

**Controlling for Non-Learners**

We identified several participants that that did not learn during the task (i.e., non-learners). These were defined by performing below <50% in the Self-condition and comprised 38/234 individuals in the lab study and 74/258 individuals in the online study. For details on these individuals, see below.

| **Supplementary Table S4A.** Ratios of non-learners in the self-condition per group | | | | |
| --- | --- | --- | --- | --- |
|  | **Lab Study** | | **Online Study** | |
| *Group* | *%* |  | *%* |  |
| AUD | 39.5 |  | 55.4 |  |
| HC | 60.5 |  | 44.6 |  |

Given that the non-learners might drive our null result, we controlled for this in an additional set of analyses. Our results demonstrated no effect of non-learners on our main results.

| **Supplementary Table S4B.** Logistic multilevel linear regression model for assessing group differences controlling for non-learners | | | | |
| --- | --- | --- | --- | --- |
|  | **Lab Study** | | **Online Study** | |
| *Predictor* | *ChiSq* | *p* | *ChiSq* | *p* |
| Group | 0.08 | .777 | 1.80 | .179 |
| Condition | 39.37 | **<.001** | 63.80 | **<.001** |
| Group:Condition | 1.18 | .553 | 0.13 | .936 |

### Controlling for Doubt in Cover Story

Basic model controlling for whether the participant expressed doubt that the interaction partner was another real participant, see Supplementary Methods for information on funnel debriefing interview.

*Model specification:*

﻿*glmer(HighProbChoice ~ Group + Condition + Doubt + Group:Condition + Group:Doubt + (1 + Condition | Participant))*

| **Supplementary Table S5.** Logistic multilevel linear regression model for assessing group differences controlling for doubt in cover story | | | | |
| --- | --- | --- | --- | --- |
|  | **Lab Study** | | **Online Study** | |
| *Predictor* | *ChiSq* | *p* | *ChiSq* | *p* |
| Group | 0.09 | .761 | 3.55 | .059 |
| Doubt | 3.01 | .082 | 4.05 | **.044** |
| Condition | 16.32 | **<.001** | 22.37 | **<.001** |
| Group: Doubt | 0.19 | .657 | 0.13 | .712 |
| Group:Condition | 0.85 | .652 | 2.94 | .229 |
| Doubt:Condition | 0.75 | .685 | 0.27 | .873 |
| Group: Doubt:Condition | 2.54 | .280 | 0.002 | .998 |


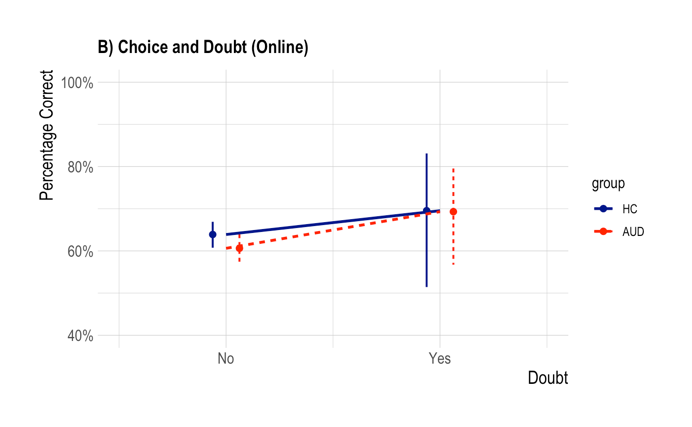

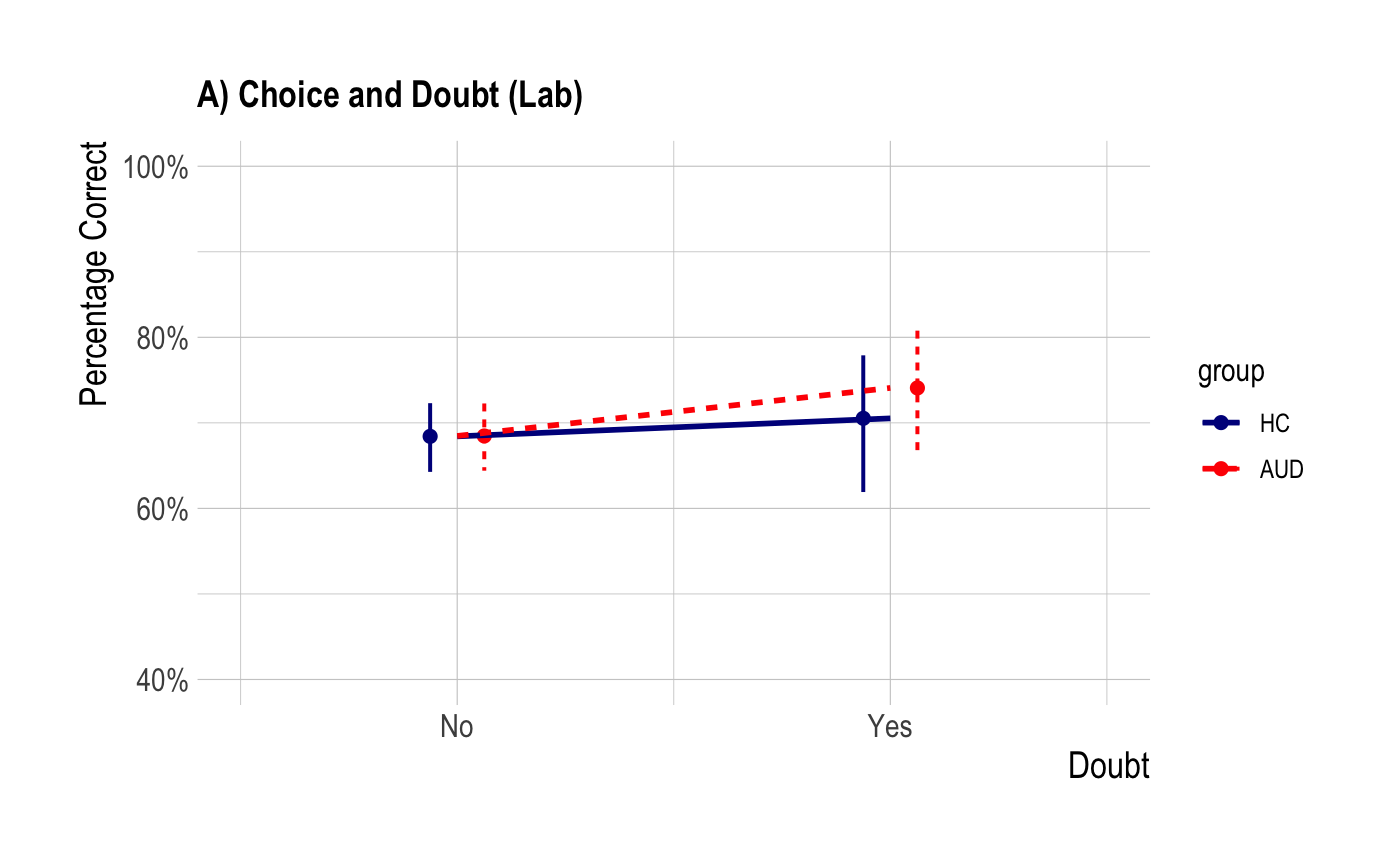


**Supplementary Figure S4.** Effect of doubt in cover story on choice averaged over conditions for the Lab (A) and Online samples (B). Effects reported in Supplementary Table S5.

### Controlling for Differences in Age

Basic models controlling for age given that it slightly differed in the online sample.

*Model specifications (Age model):*

﻿*glmer(HighProbChoice ~ Group + Condition + AgeZ + Group:Condition + Group:AgeZ + (1 + Condition | Participant))*

| **Supplementary Table S6.** Logistic multilevel linear regression model for assessing group differences in prosocial learning controlling for age. | | | | |
| --- | --- | --- | --- | --- |
|  | **Lab Study** | | **Online Study** | |
| *Predictor* | *ChiSq* | *p* | *ChiSq* | *p* |
| Group | 0.004 | .944 | 4.43 | **.035** |
| Condition | 16.23 | **<.001** | 21.33 | **<.001** |
| AgeZ | 4.07 | **.043** | 4.35 | **.036** |
| Group:Condition | 0.70 | .702 | 4.65 | .097 |
| Group:AgeZ | 0.017 | .893 | 0.97 | .323 |
| Condition:AgeZ | 0.35 | .839 | 4.54 | .103 |
| Group:Condition:AgeZ | 1.49 | .474 | 1.86 | .394 |


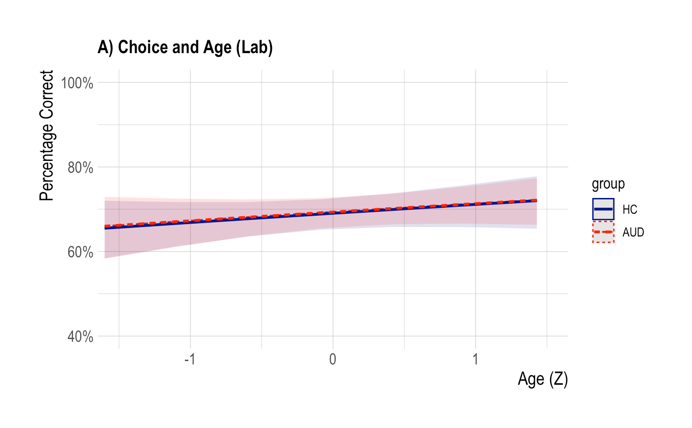

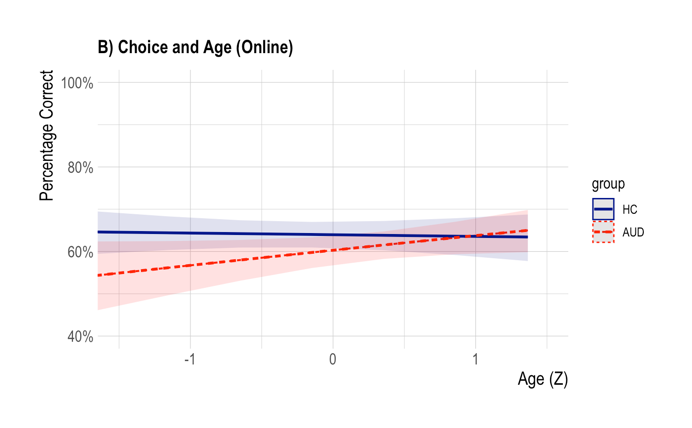


**Supplementary Figure S5.** Effect of age (z-standardized) on choice averaged over conditions for the Lab (A) and Online samples (B). Effects reported in Supplementary Table S6.

### Controlling for Differences in Socioeconomics in terms of Education

Basic models controlling for education level given that slight differences in the online sample.

*Model specifications (Education model):*

﻿*glmer(HighProbChoice ~ Group + Condition + Education + Group:Condition + Group:Education + (1 + Condition | Participant))*

| **Supplementary Table S7A.** Logistic multilevel linear regression model for assessing group differences in prosocial learning controlling for education. | | | | |
| --- | --- | --- | --- | --- |
|  | **Lab Study** | | **Online Study** | |
| *Predictor* | *ChiSq* | *p* | *ChiSq* | *p* |
| Group | 0.01 | .905 | 3.55 | .059 |
| Condition | 16.20 | **<.001** | 21.30 | **<.001** |
| Edu | 2.19 | .138 | 1.80 | .178 |
| Group:Condition | 0.72 | .695 | 3.90 | .142 |
| Group:Education | 0.67 | .410 | 1.68 | .193 |
| Condition:Education | 0.18 | .913 | 1.27 | .527 |
| Group:Condition:Education | 0.79 | .673 | 5.41 | .066 |


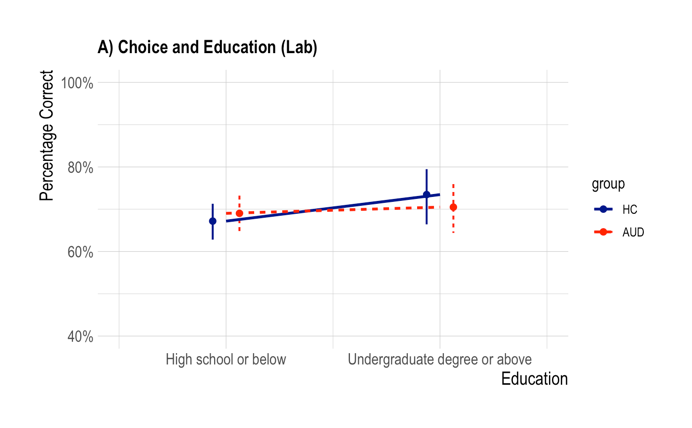

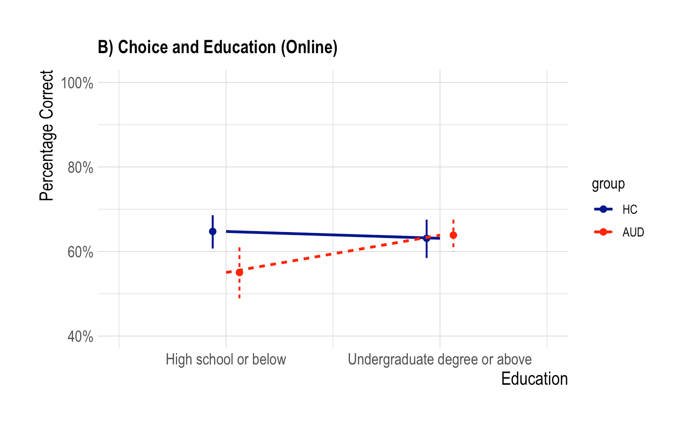


**Supplementary Figure S6.** Effect of education on choice averaged over conditions for the Lab (A) and Online samples (B). Effects reported in Supplementary Table S7.

### Controlling for Differences in Socioeconomics in terms of Income

Basic models controlling for income level given that slight differences in the online sample.

*Model specifications (Income model):*

﻿*glmer(HighProbChoice ~ Group + Condition + Income + Group:Condition + Group:Income + (1 + Condition | Participant))*

| **Supplementary Table S7B.** Logistic multilevel linear regression model for assessing group differences in prosocial learning controlling for income | | | | |
| --- | --- | --- | --- | --- |
|  | **Lab Study** | | **Online Study** | |
| *Predictor* | *ChiSq* | *p* | *ChiSq* | *p* |
| Group | 0.35 | .550 | 2.64 | .103 |
| Condition | 11.32 | **.003** | 21.32 | **<.001** |
| Income | 5.48 | **.019** | 0.18 | .668 |
| Group:Condition | 1.35 | .506 | 3.40 | .182 |
| Group:Income | 0.42 | .516 | 1.17 | .278 |
| Condition:Income | 0.50 | .778 | 1.49 | .474 |
| Group:Condition:Income | 0.36 | .834 | 5.03 | .080 |

### Controlling for Differences in Number of Close Friends

Basic models controlling for number of close friends.

*Model specifications (Income model):*

﻿*glmer(HighProbChoice ~ Group + Condition + FriendsZ + Group:Condition + Group:FriendsZ + (1 + Condition | Participant))*

| **Supplementary Table S8.** Logistic multilevel linear regression model for assessing group differences in prosocial learning controlling for number of close friends | | | | |
| --- | --- | --- | --- | --- |
|  | **Lab Study** | | **Online Study** | |
| *Predictor* | *ChiSq* | *p* | *ChiSq* | *p* |
| Group | 0.00 | .999 | 2.25 | .133 |
| Condition | 16.91 | **<.001** | 21.56 | **<.001** |
| FriendsZ | 1.01 | .315 | 5.51 | **.019** |
| Group:Condition | 1.35 | .506 | 2.96 | .228 |
| Group:FriendsZ | 0.11 | .741 | 1.00 | .317 |
| Condition:FriendsZ | 6.30 | **.043** | 2.26 | .323 |
| Group:Condition:FriendsZ | 3.75 | .153 | 4.80 | .091 |

### Controlling for Practice Effects

A basic model including only the first block per condition.

*Model specification:*

﻿*glmer(HighProbChoice ~ Group + ConditionBlock1 + Group:ConditionBlock1 + (1 + ConditionBlock1 | Participant))*

| **Supplementary Table S9.** Logistic multilevel linear regression model for assessing group differences in prosocial learning controlling for practice effects | | | | |
| --- | --- | --- | --- | --- |
|  | **Lab Study** | | **Online Study** | |
| *Predictor* | *ChiSq* | *p* | *ChiSq* | *p* |
| Group | 0.36 | .544 | 2.56 | .109 |
| Condition | 4.17 | .124 | 6.03 | **.048** |
| Group:Condition | 0.15 | .926 | 2.06 | .355 |

### Difference Score between Prosocial and Self-Condition

To validate our findings, we created a difference score by contrasting the 16*3 trial-by-trial choice response for the prosocial and self-condition. The difference score was created by subtracting the response in the self-condition from the response in the prosocial condition so that ‘0-1=-1’ corresponded to selfish response; ‘1-0=1’ to a prosocial response; and 1-1=0 or 0-0=0 to a no-difference response. The response distribution looks as follows:

##### **Supplementary Table S10A.** Response distribution for difference score between prosocial and self condition

|  | **Lab Study** | | | | **Online Study** | | | |
| --- | --- | --- | --- | --- | --- | --- | --- | --- |
| *Difference score* |  |  | |  |  |  | |  |
| -1 (Self) |  | 2358 | |  |  | 2854 | |  |
| 1 (Prosocial) |  | 2189 | |  |  | 2795 | |  |
| 0 (No difference) |  | 6573 | |  |  | 5673 | |  |
|  | | |  | | | |  | |

Next, we investigated group differences in difference score using the following model specification:

*Model specification:*

*lmer(DifferenceScore ~ Group + (1 | Participant))*

| **Supplementary Table S10B.** Multilevel linear regression model for assessing group differences in difference score between learning in the prosocial and self-condition of the learning task. | | | | |
| --- | --- | --- | --- | --- |
|  | **Lab Study** | | **Online Study** | |
| *Predictor* | *ChiSq* | *p* | *ChiSq* | *p* |
| Group | 0.27 | .598 | 0.50 | .477 |

### Exchanging the Group Variable for Self-Reported Alcohol Use Past Year: AUDIT

To validate the impact of alcohol use disorder on prosociality, we switched the group-variable (i.e., AUD vs. HC) for a continues variable from a self-report measure on Alcohol Use Disorder (AUDIT).

*Model specification:*

*glmer(HighProbChoice ~ AUDITZ + Condition + AUDITZ:Condition + (1 + Condition | Participant))*

| **Supplementary Table S11.** Logistic multilevel linear regression model for assessing the relation between AUDIT and prosocial learning | | | | |
| --- | --- | --- | --- | --- |
|  | **Lab Study** | | **Online Study** | |
| *Predictor* | *ChiSq* | *p* | *ChiSq* | *p* |
| AUDITZ | 0.35 | .554 | 4.04 | **.044** |
| Condition | 16.18 | **<.001** | 21.35 | **<.001** |
| AUDITZ:Condition | 1.65 | .436 | 1.36 | .504 |


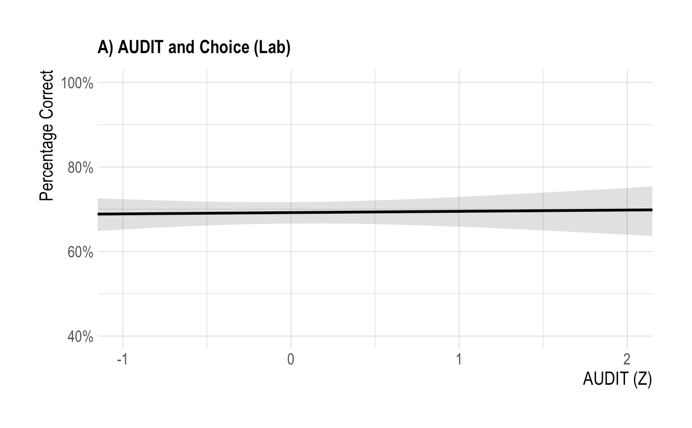

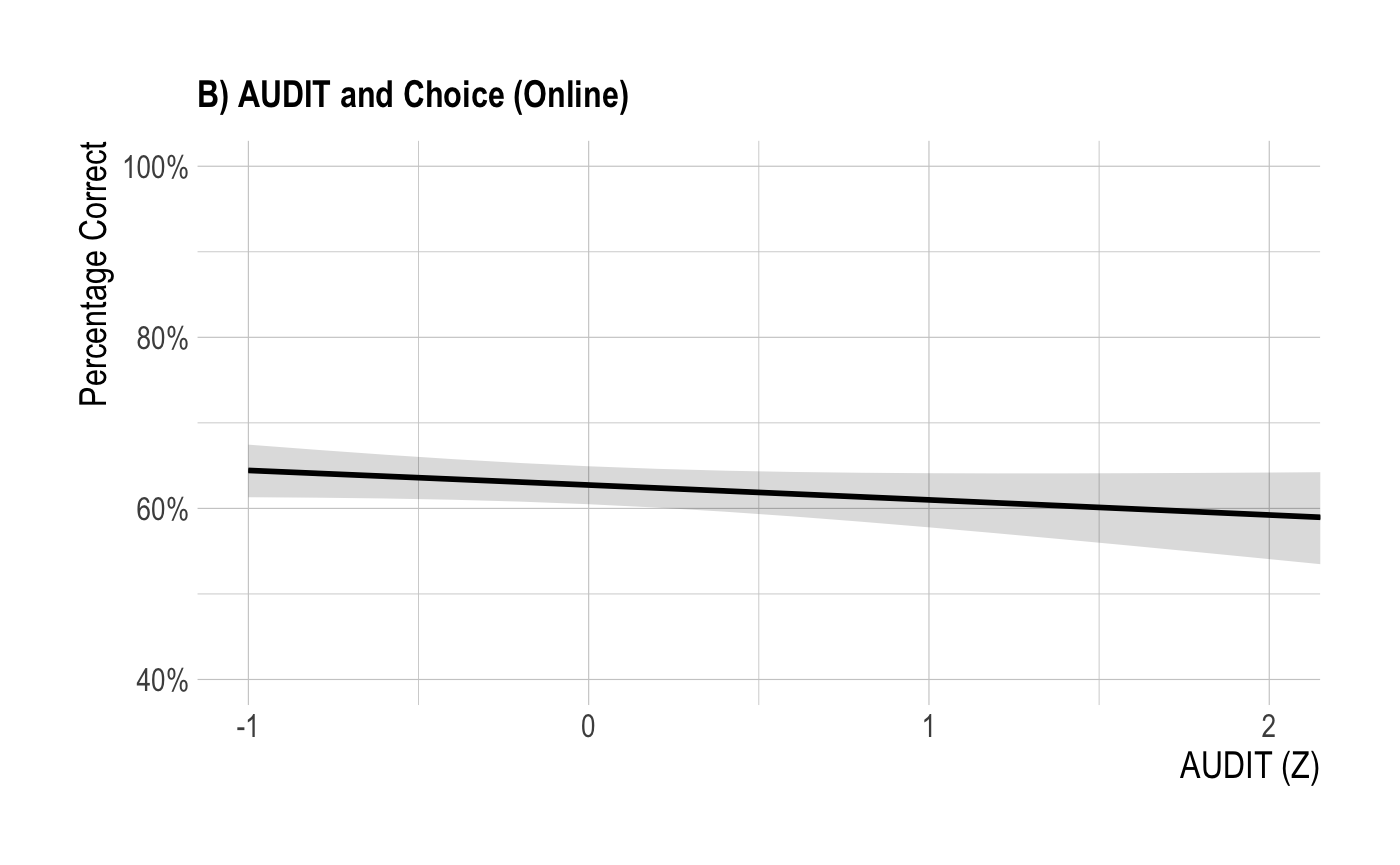


**Supplementary Figure S7.** Effect of AUDIT on choice averaged across conditions for the Lab (A) and Online samples (B). Effects reported in Supplementary Table S11.

### Exchanging the Group Variable for Fulfilled AUD Criteria

To validate the impact of alcohol use disorder on prosociality, we switched the group-variable (i.e., AUD vs. HC) for a continuous variable on a number of fulfilled criteria for AUD.

*Model specification:*

*glmer(HighProbChoice ~ AUDCriteriaZ + Condition + AUDCriteriaZ:Condition + (1 + Condition | Participant))*

| **Supplementary Table S12A.** Logistic multilevel linear regression model for assessing the relation between AUD criteria and prosocial learning | | | | |
| --- | --- | --- | --- | --- |
|  | **Lab Study** | | **Online Study** | |
| *Predictor* | *ChiSq* | *p* | *ChiSq* | *p* |
| AUD.CriteriaZ | 1.81 | .177 | 3.58 | .058 |
| Condition | 8.69 | **.013** | 21.33 | **<.001** |
| AUD.CriteriaZ:Condition | 0.86 | .649 | 2.70 | .258 |


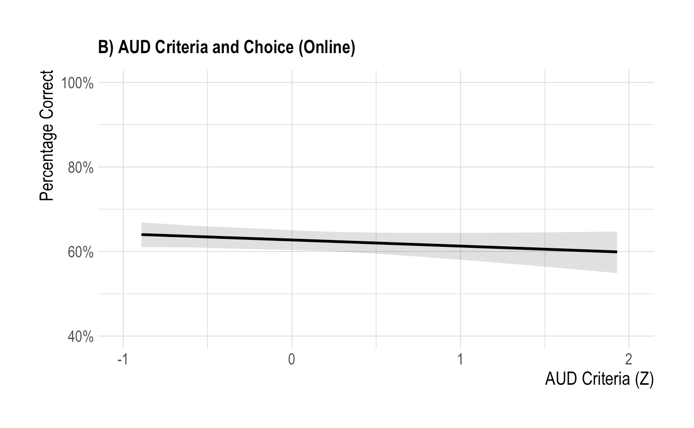


**Supplementary Figure S8.** Effect of AUD criteria on choice averaged across conditions for the Lab (A) and Online samples (B). Effects reported in Supplementary Table S12A.


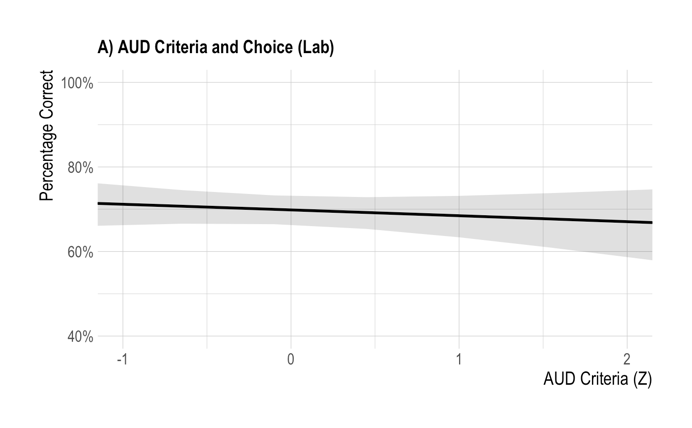


### Exchanging the Group Variable for Fulfilled ‘Social’ AUD Criteria

Furthermore, we assessed the social criteria for AUD (i.e., the criteria accounting for negative social consequences of alcohol use).

*Model specification:*

*glmer(HighProbChoice ~ AUDCriteriaSocZ + Condition + AUDCriteriaSocZ:Condition + (1 + Condition | Participant))*

| **Supplementary Table S12B** Logistic multilevel linear regression model for assessing the relation between social AUD criteria and prosocial learning | | | | |
| --- | --- | --- | --- | --- |
|  | **Lab Study** | | **Online Study** | |
| *Predictor* | *ChiSq* | *p* | *ChiSq* | *p* |
| AUD.CriteriaSocZ | 0.06 | .802 | 3.09 | .078 |
| Condition | 8.64 | **.013** | 21.36 | **<.001** |
| AUD.CriteriaSocZ:Condition | 0.35 | .835 | 1.13 | .567 |


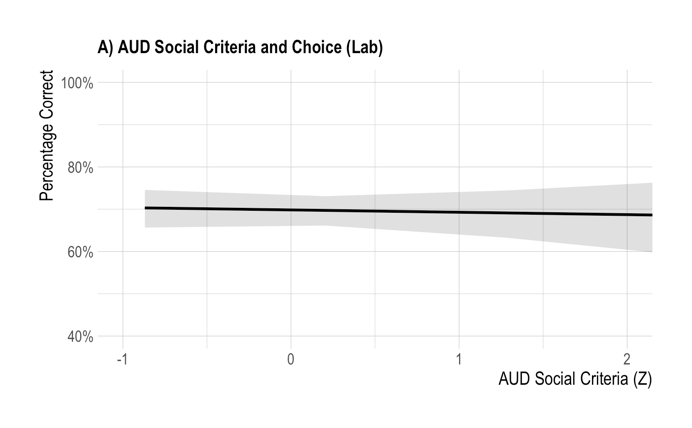

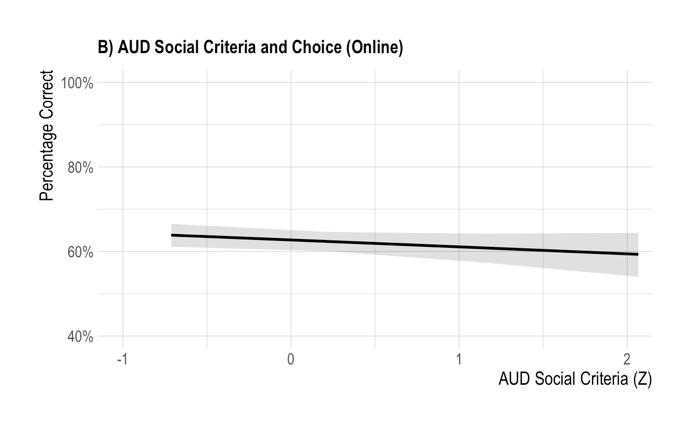


**Supplementary Figure S9.** Effect of AUD social criteria on choice averaged across conditions for the Lab (A) and Online samples (B). Effects reported in Supplementary Table S12B.

## Models Complementing the Group Variable with Additional Psychiatric Processes

Given that a diverse set of alcohol-related variables and psychiatric symptoms are commonly associated with AUD, we applied a set of additional models to assess the complementary role of alcohol craving, days of abstinence, alcohol use past 30 days, harmful drinking history, anxiety, depression, stress, as well as symptoms of ADHD and autism (see Methods in the main manuscript for details).

### Alcohol Craving

*Model specification:*

﻿*glmer(HighProbChoice ~ Group + Condition + AlcCravingZ + Group:Condition + Group:AlcCravingZ + Condition:AlcCravingZ + Group:Condition:AlcCravingZ + (1 + Condition | Participant))*

| **Supplementary Table S13.** Logistic multilevel linear regression model for assessing the relation between alcohol craving and group differences in prosocial learning | | | | |
| --- | --- | --- | --- | --- |
|  | **Lab Study** | | **Online Study** | |
| *Predictor* | *ChiSq* | *p* | *ChiSq* | *p* |
| Group | 0.23 | .630 | 0.26 | .603 |
| AlcCravingZ | 0.12 | .727 | 0.40 | .522 |
| Condition | 17.90 | **<.001** | 21.24 | **<.001** |
| Group:AlcCravingZ | 0.18 | .666 | 0.01 | .898 |
| Group:Condition | 0.46 | .794 | 2.50 | .285 |
| AlcCravingZ:Condition | 2.16 | .338 | 1.71 | .425 |
| Group:AlcCravingZ:Condition | 0.06 | .966 | 3.002 | .222 |

### Days of Abstinence

*Model specification:*

*glmer(HighProbChoice ~ Group + Condition + AlcAbstinenceZ + Group:Condition + Group:AlcAbstinenceZ + Condition:AlcAbstinenceZ + Group:Condition:AlcAbstinenceZ + (1 + Condition | Participant))*

| **Supplementary Table S14A.** Logistic multilevel linear regression model for assessing the relation between days of abstinence and group differences in prosocial learning | | | | |
| --- | --- | --- | --- | --- |
|  | **Lab Study** | | **Online Study** | |
| *Predictor* | *ChiSq* | *p* | *ChiSq* | *p* |
| Group | 0.43 | .511 | 0.26 | .607 |
| AlcAbstZ | 0.98 | .321 | 0.51 | .472 |
| Condition | 16.18 | **<.001** | 21.93 | **<.001** |
| Group:AlcAbstZ | 0.03 | .862 | 1.25 | .262 |
| Group:Condition | 0.02 | .988 | 7.82 | **.020** |
| AlcAbstZ:Condition | 0.29 | .861 | 6.25 | **.043** |
| Group: AlcAbstZ:Condition | 2.00 | .367 | 0.66 | .718 |

Abstinence indicates some type of two-way interaction effect in the online study We therefor conducted a pairwise analysis to understand this better. To limit the number of comparisons, and due to the additional role of recent alcohol consumption being our main interest, we chose to hold the abstinence variable constant at -1 standard deviation from the mean.

*Specification of analysis:*

*emmeans(model, specs = ~ Group * Condition | AlcAbstZ, at = list(AlcAbstZ = c(-1)))*

Supplementary Table S14B. Pairwise Comparisons of Alcohol abstinence at -1SD (Online Study)

| contrast | AbstinenceZ | estimate | SE | df | asymp.LCL | asymp.UCL | z.ratio | p.value |
| --- | --- | --- | --- | --- | --- | --- | --- | --- |
| HC OTHER - AUD OTHER | -1 | 0.0536 | 0.1677 | Inf | -0.4244 | 0.5316 | 0.3197 | 0.9996 |
| HC OTHER - HC NO ONE | -1 | 0.0704 | 0.1215 | Inf | -0.2758 | 0.4166 | 0.5794 | 0.9924 |
| HC OTHER - AUD NO ONE | -1 | 0.1804 | 0.1617 | Inf | -0.2804 | 0.6412 | 1.1157 | 0.8751 |
| HC OTHER - HC SELF | -1 | -0.2726 | 0.1021 | Inf | -0.5635 | 0.0182 | -2.6710 | 0.0811 |
| HC OTHER - AUD SELF | -1 | 0.0157 | 0.1688 | Inf | -0.4654 | 0.4967 | 0.0929 | 1.0000 |
| AUD OTHER - HC NO ONE | -1 | 0.0168 | 0.1534 | Inf | -0.4205 | 0.4540 | 0.1093 | 1.0000 |
| AUD OTHER - AUD NO ONE | -1 | 0.1268 | 0.0781 | Inf | -0.0958 | 0.3493 | 1.6233 | 0.5830 |
| AUD OTHER - HC SELF | -1 | -0.3262 | 0.1710 | Inf | -0.8135 | 0.1610 | -1.9080 | 0.3971 |
| AUD OTHER - AUD SELF | -1 | -0.0380 | 0.0650 | Inf | -0.2231 | 0.1472 | -0.5842 | 0.9921 |
| HC NO ONE - AUD NO ONE | -1 | 0.1100 | 0.1467 | Inf | -0.3081 | 0.5281 | 0.7497 | 0.9756 |
| HC NO ONE - HC SELF | -1 | -0.3430 | 0.1315 | Inf | -0.7178 | 0.0317 | -2.6083 | 0.0952 |
| HC NO ONE - AUD SELF | -1 | -0.0547 | 0.1545 | Inf | -0.4951 | 0.3857 | -0.3541 | 0.9993 |
| AUD NO ONE - HC SELF | -1 | -0.4530 | 0.1650 | Inf | -0.9232 | 0.0172 | -2.7453 | 0.0667 |
| AUD NO ONE - AUD SELF | -1 | -0.1647 | 0.0840 | Inf | -0.4040 | 0.0745 | -1.9619 | 0.3645 |
| HC SELF - AUD SELF | -1 | 0.2883 | 0.1719 | Inf | -0.2017 | 0.7782 | 1.6768 | 0.5472 |

### Alcohol Use in the Past 30 days


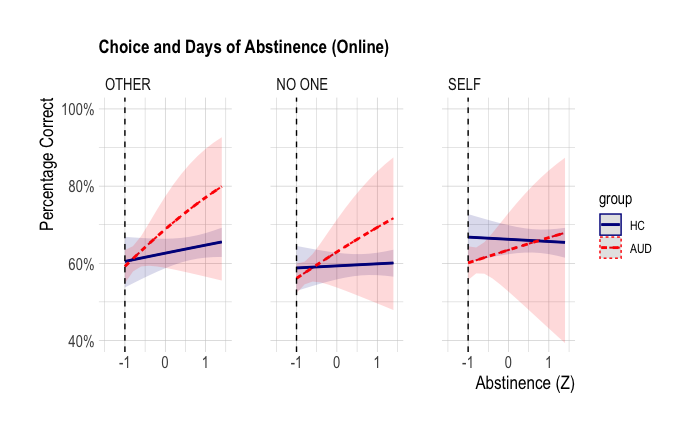


**Supplementary Figure S10.** Effect of days of abstinence on choice separated by condition for the Online sample. Effects are reported in Supplementary Table S14B.

*Model specification:*

*glmer(HighProbChoice ~ Group + Condition + TLFB30Z + Group:Condition + Group:TLFB30Z + Condition:TLFB30Z + Group:Condition:TLFB30Z + (1 + Condition | Participant))*

| **Supplementary Table S15A.** Logistic multilevel linear regression model for assessing the relation between past alcohol use and group differences in prosocial learning | | | | |
| --- | --- | --- | --- | --- |
|  | **Lab Study** | | **Online Study** | |
| *Predictor* | *ChiSq* | *p* | *ChiSq* | *p* |
| Group | 1.11 | .290 | 0.05 | .821 |
| TLFB30Z | 2.26 | .132 | 1.14 | .285 |
| Condition | 16.53 | **<.001** | 21.19 | **<.001** |
| Group:TLFB30Z | 4.03 | **.044** | 0.20 | .651 |
| Group:Condition | 0.95 | .621 | 2.77 | .249 |
| TLFB30Z:Condition | 0.27 | .873 | 1.69 | .427 |
| Group:TLFB30Z:Condition | 1.54 | .462 | 10.96 | **.004** |

Alcohol use past 30 days indicate some type of two or three-way interaction effect both the lab and online study. We therefor conducted a pairwise analysis to understand this better. To avoid to many pairwise comparisons, and due to additional alcohol use past 30 days being our main interest, we chose to hold the duration of the alcohol use past 30 days variable constant at +1 standard deviation from the mean.

*Specification of analysis:*

*emmeans(model, specs = ~ Group * Condition | TLFB30Z, at = list(TLFB30Z = c(1)))*

Supplementary Table S15B. Pairwise Comparisons of Alcohol use past 30 days at +1SD (Lab Study)

| contrast | TLFB30Z | estimate | SE | df | asymp.LCL | asymp.UCL | z.ratio | p.value |
| --- | --- | --- | --- | --- | --- | --- | --- | --- |
| HC OTHER - AUD OTHER | 1 | 2.068 | 1.4336 | Inf | -2.0178 | 6.1528 | 1.442 | 0.7011 |
| HC OTHER - HC NO ONE | 1 | -0.203 | 1.2075 | Inf | -3.6437 | 3.2384 | -0.168 | 1.0000 |
| HC OTHER - AUD NO ONE | 1 | 2.237 | 1.4328 | Inf | -1.8457 | 6.3205 | 1.562 | 0.6241 |
| HC OTHER - HC SELF | 1 | -1.607 | 1.3201 | Inf | -5.3693 | 2.1544 | -1.218 | 0.8284 |
| HC OTHER - AUD SELF | 1 | 1.956 | 1.4337 | Inf | -2.1294 | 6.0418 | 1.364 | 0.7484 |
| AUD OTHER - HC NO ONE | 1 | -2.270 | 1.3577 | Inf | -6.1391 | 1.5988 | -1.672 | 0.5504 |
| AUD OTHER - AUD NO ONE | 1 | 0.170 | 0.0727 | Inf | -0.0372 | 0.3769 | 2.338 | 0.1789 |
| AUD OTHER - HC SELF | 1 | -3.675 | 1.6012 | Inf | -8.2379 | 0.8880 | -2.295 | 0.1959 |
| AUD OTHER - AUD SELF | 1 | -0.111 | 0.0761 | Inf | -0.3283 | 0.1057 | -1.462 | 0.6889 |
| HC NO ONE - AUD NO ONE | 1 | 2.440 | 1.3568 | Inf | -1.4266 | 6.3067 | 1.798 | 0.4668 |
| HC NO ONE - HC SELF | 1 | -1.405 | 1.5321 | Inf | -5.7708 | 2.9613 | -0.917 | 0.9423 |
| HC NO ONE - AUD SELF | 1 | 2.159 | 1.3578 | Inf | -1.7105 | 6.0283 | 1.590 | 0.6052 |
| AUD NO ONE - HC SELF | 1 | -3.845 | 1.6006 | Inf | -8.4060 | 0.7164 | -2.402 | 0.1554 |
| **AUD NO ONE - AUD SELF** | **1** | **-0.281** | **0.0869** | **Inf** | **-0.5287** | **-0.0336** | **-3.237** | **0.0153** |
| HC SELF - AUD SELF | 1 | 3.564 | 1.6014 | Inf | -1.0000 | 8.1273 | 2.225 | 0.2260 |


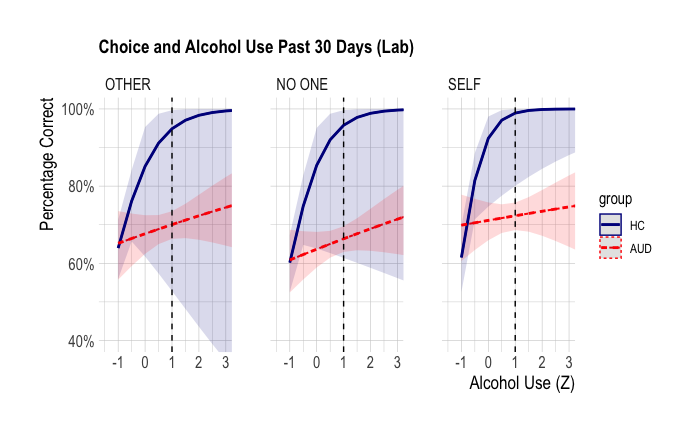


**Supplementary Figure S11.** Effect of alcohol use in the past 30 days on choice separated by condition for the Lab sample. Effects are reported in Supplementary Table S15B.

Supplementary Table S15C. Pairwise Comparisons of Alcohol use past 30 days at +1SD (Online Study)

| contrast | TLFB30Z | estimate | SE | df | asymp.LCL | asymp.UCL | z.ratio | p.value |
| --- | --- | --- | --- | --- | --- | --- | --- | --- |
| HC OTHER - AUD OTHER | 1 | -1.7055 | 0.9206 | Inf | -4.3290 | 0.9181 | -1.8525 | 0.4319 |
| HC OTHER - HC NO ONE | 1 | -1.7214 | 0.7885 | Inf | -3.9684 | 0.5256 | -2.1831 | 0.2456 |
| HC OTHER - AUD NO ONE | 1 | -1.5665 | 0.9200 | Inf | -4.1883 | 1.0554 | -1.7026 | 0.5300 |
| **HC OTHER - HC SELF** | **1** | **-2.1816** | **0.7175** | **Inf** | **-4.2264** | **-0.1369** | **-3.0405** | **0.0285** |
| HC OTHER - AUD SELF | 1 | -1.6868 | 0.9208 | Inf | -4.3107 | 0.9372 | -1.8319 | 0.4450 |
| AUD OTHER - HC NO ONE | 1 | -0.0159 | 0.8804 | Inf | -2.5249 | 2.4930 | -0.0181 | 1.0000 |
| AUD OTHER - AUD NO ONE | 1 | 0.1390 | 0.0607 | Inf | -0.0341 | 0.3121 | 2.2887 | 0.1985 |
| AUD OTHER - HC SELF | 1 | -0.4762 | 1.0238 | Inf | -3.3938 | 2.4414 | -0.4651 | 0.9973 |
| AUD OTHER - AUD SELF | 1 | 0.0187 | 0.0511 | Inf | -0.1269 | 0.1643 | 0.3658 | 0.9991 |
| HC NO ONE - AUD NO ONE | 1 | 0.1549 | 0.8798 | Inf | -2.3522 | 2.6621 | 0.1761 | 1.0000 |
| HC NO ONE - HC SELF | 1 | -0.4603 | 0.9398 | Inf | -3.1385 | 2.2180 | -0.4897 | 0.9965 |
| HC NO ONE - AUD SELF | 1 | 0.0346 | 0.8806 | Inf | -2.4748 | 2.5440 | 0.0393 | 1.0000 |
| AUD NO ONE - HC SELF | 1 | -0.6152 | 1.0233 | Inf | -3.5313 | 2.3009 | -0.6012 | 0.9910 |
| AUD NO ONE - AUD SELF | 1 | -0.1203 | 0.0660 | Inf | -0.3083 | 0.0677 | -1.8236 | 0.4504 |
| HC SELF - AUD SELF | 1 | 0.4949 | 1.0239 | Inf | -2.4230 | 3.4128 | 0.4833 | 0.9968 |


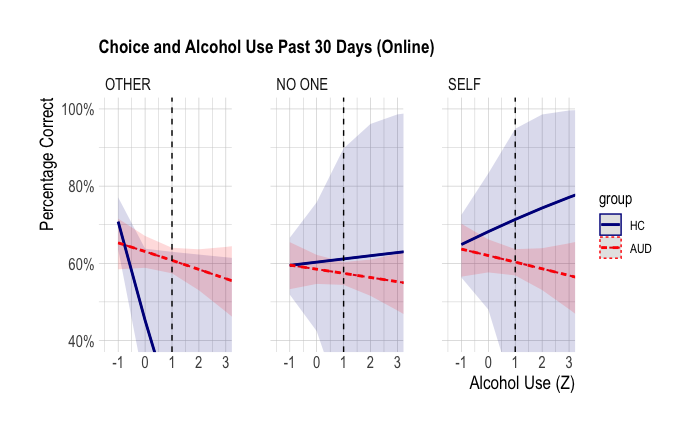


**Supplementary Figure S12.** Effect of alcohol use in the past 30 days on choice separated by condition for the Online sample. Effects are reported in Supplementary Table S15C.

### Harmful Drinking History

*Model specification:*

*glmer(HighProbChoice ~ Group + Condition + DurHarmDrinkingZ + Group:Condition + Group:DurHarmDrinkingZ + Condition:DurHarmDrinkingZ + Group:Condition:DurHarmDrinkingZ + (1 + Condition | Participant))*

| **Supplementary Table S16A.** Logistic multilevel linear regression model for assessing the relation between harmful drinking history and group differences in prosocial learning | | | | |
| --- | --- | --- | --- | --- |
|  | **Lab Study** | | **Online Study** | |
| *Predictor* | *ChiSq* | *p* | *ChiSq* | *p* |
| Group | 0.14 | .702 | 1.58 | .208 |
| DurHarmDrinkingZ | 0.99 | .317 | 0.001 | .973 |
| Condition | 16.24 | **<.001** | 21.34 | **<.001** |
| Group: DurHarmDrinkingZ | 0.63 | .424 | 0.95 | .328 |
| Group:Condition | 1.95 | .375 | 1.12 | .570 |
| DurHarmDrinkingZ:Condition | 1.27 | .527 | 2.20 | .332 |
| Group: DurHarmDrinkingZ:Condition | 6.85 | **.032** | 0.54 | .760 |

Duration of harmful drinking indicate some type of three-way interaction effect in the lab study. We therefor conducted a pairwise analysis to understand this better. To avoid to many pairwise comparisons, and due to additional alcohol use symptoms being our main interest, we chose to hold the duration of harmful drinking variable constant at +1 standard deviation from the mean.

*Specification of analysis:*

*emmeans(model, specs = ~ Group * Condition | DurHarmDrinkingZ, at = list(DurHarmDrinkingZ = c(1)))*

Supplementary Table S16B. Pairwise Comparisons of harmful drinking history at +1SD (Lab Study)

| contrast | HarmfulDrinkingZ | estimate | SE | df | asymp.LCL | asymp.UCL | z.ratio | p.value |
| --- | --- | --- | --- | --- | --- | --- | --- | --- |
| HC OTHER - AUD OTHER | 1 | -1.6736 | 0.8211 | Inf | -4.013 | 0.666163 | -2.038 | 0.3204 |
| HC OTHER - HC NO ONE | 1 | -1.4108 | 0.6786 | Inf | -3.345 | 0.523019 | -2.079 | 0.2983 |
| HC OTHER - AUD NO ONE | 1 | -1.5088 | 0.8199 | Inf | -3.845 | 0.827584 | -1.840 | 0.4397 |
| HC OTHER - HC SELF | 1 | -1.2893 | 0.6990 | Inf | -3.281 | 0.702532 | -1.845 | 0.4369 |
| HC OTHER - AUD SELF | 1 | -1.7638 | 0.8215 | Inf | -4.105 | 0.577312 | -2.147 | 0.2632 |
| AUD OTHER - HC NO ONE | 1 | 0.2629 | 0.7397 | Inf | -1.845 | 2.370889 | 0.355 | 0.9993 |
| AUD OTHER - AUD NO ONE | 1 | 0.1649 | 0.0740 | Inf | -0.046 | 0.375807 | 2.228 | 0.2249 |
| AUD OTHER - HC SELF | 1 | 0.3844 | 0.8768 | Inf | -2.114 | 2.882951 | 0.438 | 0.9980 |
| AUD OTHER - AUD SELF | 1 | -0.0902 | 0.0778 | Inf | -0.312 | 0.131523 | -1.159 | 0.8562 |
| HC NO ONE - AUD NO ONE | 1 | -0.0980 | 0.7385 | Inf | -2.202 | 2.006446 | -0.133 | 1.0000 |
| HC NO ONE - HC SELF | 1 | 0.1215 | 0.8255 | Inf | -2.231 | 2.473850 | 0.147 | 1.0000 |
| HC NO ONE - AUD SELF | 1 | -0.3530 | 0.7403 | Inf | -2.463 | 1.756705 | -0.477 | 0.9970 |
| AUD NO ONE - HC SELF | 1 | 0.2195 | 0.8757 | Inf | -2.276 | 2.715070 | 0.251 | 0.9999 |
| **AUD NO ONE - AUD SELF** | **1** | **-0.2551** | **0.0895** | **Inf** | **-0.510** | **-0.000158** | **-2.851** | **0.0498** |
| HC SELF - AUD SELF | 1 | -0.4745 | 0.8774 | Inf | -2.975 | 2.025678 | -0.541 | 0.9945 |


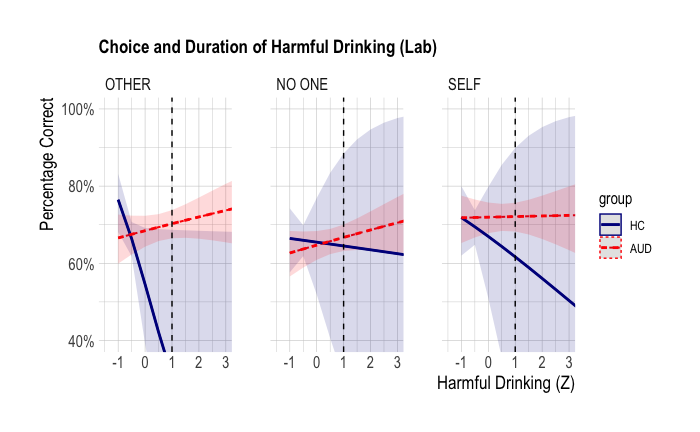


**Supplementary Figure S13.** Effect of duration of harmful drinking on choice separated by condition for the Lab sample. Effects are reported in Supplementary Table S16B.

### Anxiety Symptoms

*Model specification:*

*glmer(HighProbChoice ~ DASS21.AnxietyZ + Condition + Group + DASS21.AnxietyZ:Condition + DASS21.AnxietyZ:Condition:Group + (1 + Condition | Participant))*

| **Supplementary Table S17A.** Logistic multilevel linear regression model for assessing the relation between anxiety symptoms and group differences in prosocial learning | | | | |
| --- | --- | --- | --- | --- |
|  | **Lab Study** | | **Online Study** | |
| *Predictor* | *ChiSq* | *p* | *ChiSq* | *p* |
| Group | 0.02 | .867 | 1.69 | .193 |
| DASS21.AnxietyZ | 0.04 | .823 | 0.20 | .654 |
| Condition | 16.69 | **<.001** | 21.39 | **<.001** |
| Group:DASS21.AnxietyZ | 0.43 | .509 | 2.32 | .127 |
| Group:Condition | 0.23 | .889 | 4.01 | .134 |
| DASS21.AnxietyZ:Condition | 1.49 | .474 | 3.14 | .207 |
| Group:DASS21.AnxietyZ:Condition | 9.48 | **.008** | 1.30 | .520 |

Symptoms of anxiety indicate some type of three-way interaction effect in the lab study. We therefor conducted a pairwise analysis to understand this better. To limit the number of comparisons, and due to the additional role of increased psychiatric symptoms being our main interest, we chose to hold the anxiety symptom variable constant at +1 standard deviation from the mean.

*Specification of analysis:*

*emmeans(model, specs = ~ Group * Condition | DASS21.AnxietyZ, at = list(DASS21. AnxietyZ = c(1)))*

Supplementary Table S17B. Pairwise Comparisons of Anxiety symptoms at +1SD (Lab Study)

| contrast | AnxietyZ | estimate | SE | df | asymp.LCL | asymp.UCL | z.ratio | p.value |
| --- | --- | --- | --- | --- | --- | --- | --- | --- |
| HC OTHER - AUD OTHER | 1 | 0.3529 | 0.2526 | Inf | -0.3669 | 1.0728 | 1.397 | 0.7288 |
| **HC OTHER - HC NO ONE** | **1** | **0.5754** | **0.1922** | **Inf** | **0.0276** | **1.1232** | **2.993** | **0.0329** |
| HC OTHER - AUD NO ONE | 1 | 0.4769 | 0.2489 | Inf | -0.2324 | 1.1862 | 1.916 | 0.3923 |
| HC OTHER - HC SELF | 1 | -0.1960 | 0.2093 | Inf | -0.7924 | 0.4004 | -0.936 | 0.9371 |
| HC OTHER - AUD SELF | 1 | 0.1977 | 0.2544 | Inf | -0.5274 | 0.9227 | 0.777 | 0.9714 |
| AUD OTHER - HC NO ONE | 1 | 0.2225 | 0.2246 | Inf | -0.4175 | 0.8624 | 0.991 | 0.9211 |
| AUD OTHER - AUD NO ONE | 1 | 0.1239 | 0.0771 | Inf | -0.0959 | 0.3438 | 1.606 | 0.5942 |
| AUD OTHER - HC SELF | 1 | -0.5489 | 0.2642 | Inf | -1.3018 | 0.2040 | -2.078 | 0.2990 |
| AUD OTHER - AUD SELF | 1 | -0.1553 | 0.0818 | Inf | -0.3883 | 0.0778 | -1.898 | 0.4030 |
| HC NO ONE - AUD NO ONE | 1 | -0.0985 | 0.2201 | Inf | -0.7258 | 0.5288 | -0.448 | 0.9977 |
| **HC NO ONE - HC SELF** | **1** | **-0.7714** | **0.2316** | **Inf** | **-1.4314** | **-0.1114** | **-3.330** | **0.0112** |
| HC NO ONE - AUD SELF | 1 | -0.3777 | 0.2264 | Inf | -1.0228 | 0.2674 | -1.668 | 0.5528 |
| AUD NO ONE - HC SELF | 1 | -0.6729 | 0.2605 | Inf | -1.4151 | 0.0694 | -2.583 | 0.1013 |
| **AUD NO ONE - AUD SELF** | **1** | **-0.2792** | **0.0926** | **Inf** | **-0.5432** | **-0.0152** | **-3.014** | **0.0309** |
| HC SELF - AUD SELF | 1 | 0.3937 | 0.2655 | Inf | -0.3630 | 1.1503 | 1.483 | 0.6755 |


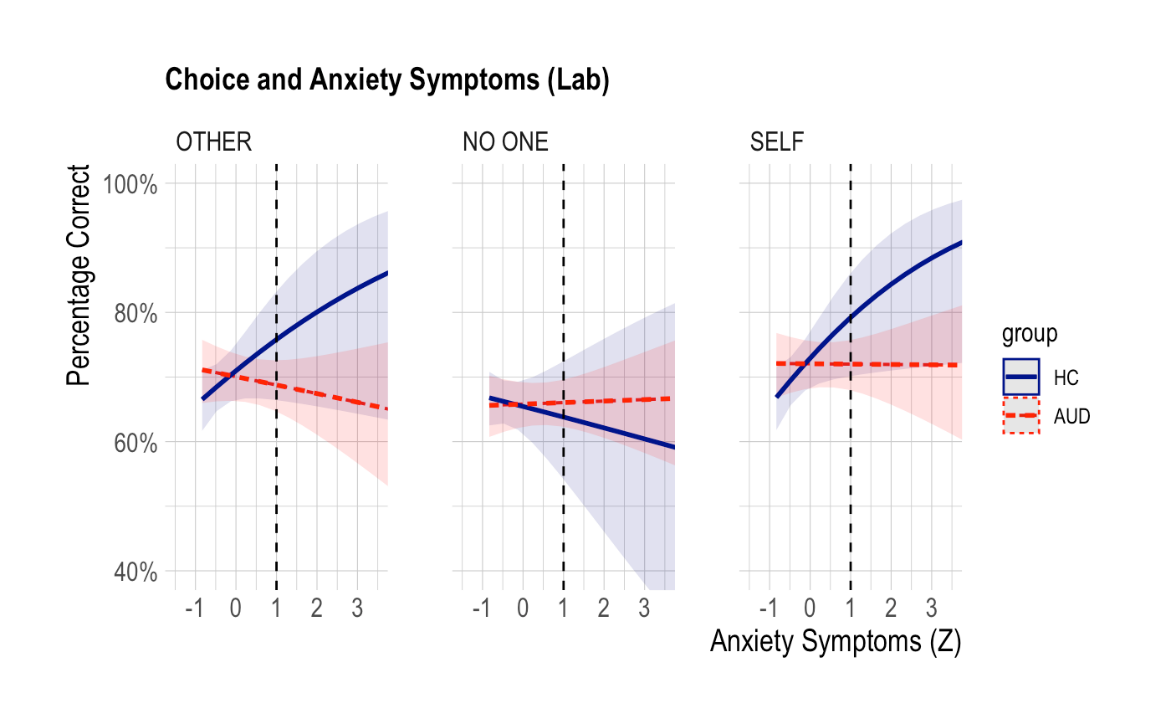


**Supplementary Figure S14.** Effect of anxiety symptoms on choice separated by condition for the Lab sample. Effects are reported in Supplementary Table S17B.

### Depression Symptoms

*Model specification:*

*glmer(HighProbChoice ~ DASS21.DepressionZ + Condition + Group + DASS21. DepressionZ:Condition + DASS21.DepressionZ:Condition:Group + (1 + Condition | Participant))*

| **Supplementary Table S18A.** Logistic multilevel linear regression model for assessing the relation between depression symptoms and group differences in prosocial learning | | | | |
| --- | --- | --- | --- | --- |
|  | **Lab Study** | | **Online Study** | |
| *Predictor* | *ChiSq* | *p* | *ChiSq* | *p* |
| Group | 0.0009 | .975 | 4.33 | **.037** |
| DASS21.DepressionZ | 0.19 | .659 | 2.91 | .087 |
| Condition | 16.42 | **<.001** | 21.50 | **<.001** |
| Group:DASS21.DepressionZ | 0.59 | .440 | 0.03 | .847 |
| Group:Condition | 0.73 | .693 | 4.58 | .100 |
| DASS21.DepressionZ:Condition | 1.91 | .382 | 2.15 | .340 |
| Group:DASS21.DepressionZ:Condition | 2.57 | .275 | 5.55 | .062 |

### Stress Symptoms

*Model specification:*

*glmer(HighProbChoice ~ DASS21.StressZ + Condition + Group + DASS21. StressZ:Condition + DASS21.StressZ:Condition:Group + (1 + Condition | Participant))*

| **Supplementary Table S19A.** Logistic multilevel linear regression model for assessing the relation between stress symptoms and group differences in prosocial learning | | | | |
| --- | --- | --- | --- | --- |
|  | **Lab Study** | | **Online Study** | |
| *Predictor* | *ChiSq* | *p* | *ChiSq* | *p* |
| Group | 0.00 | .995 | 2.77 | .095 |
| DASS21.StressZ | 0.43 | .509 | 0.21 | .640 |
| Condition | 16.37 | **<.001** | 21.42 | **<.001** |
| Group:DASS21.StressZ | 0.53 | .464 | 2.32 | .127 |
| Group:Condition | 0.97 | .613 | 4.34 | .113 |
| DASS21.StressZ:Condition | 4.93 | .084 | 1.81 | .403 |
| Group:DASS21.StressZ:Condition | 4.03 | .132 | 3.11 | .210 |

### ADHD Symptoms

*Model specification:*

*glmer(HighProbChoice ~ ASRSZ + Condition + Group + ASRSZ:Condition + ASRSZ:Condition:Group + (1 + Condition | Participant))*

| **Supplementary Table S20A.** Logistic multilevel linear regression model for assessing the relation between ADHD symptoms and group differences in prosocial learning | | | | |
| --- | --- | --- | --- | --- |
|  | **Lab Study** | | **Online Study** | |
| *Predictor* | *ChiSq* | *p* | *ChiSq* | *p* |
| Group | 0.12 | .721 | 2.19 | .138 |
| ASRSZ | 0.07 | .786 | 0.63 | .425 |
| Condition | 16.38 | **<.001** | 21.10 | **<.001** |
| Group: ASRSZ | 0.21 | .640 | 0.85 | .353 |
| Group:Condition | 0.19 | .909 | 4.77 | .091 |
| ASRSZ:Condition | 2.64 | .266 | 4.46 | .107 |
| Group: ASRSZ:Condition | 1.01 | .601 | 2.46 | .292 |

### Autism Symptoms

*Model specification:*

*glmer(HighProbChoice ~ AQ10Z + Condition + Group + AQ10Z:Condition + AQ10Z:Condition:Group + (1 + Condition | Participant))*

| **Supplementary Table S21.** Logistic multilevel linear regression model for assessing the relation between autism symptoms and group differences in prosocial learning | | | | |
| --- | --- | --- | --- | --- |
|  | **Lab Study** | | **Online Study** | |
| *Predictor* | *ChiSq* | *p* | *ChiSq* | *p* |
| Group | 0.13 | .712 | 2.27 | .131 |
| AQ.10Z | 1.56 | .211 | 0.38 | .537 |
| Condition | 16.15 | **<.001** | 21.42 | **<.001** |
| Group:AQ.10Z | 0.74 | .387 | 0.96 | .325 |
| Group:Condition | 1.02 | .598 | 2.94 | .229 |
| AQ.10Z:Condition | 2.61 | .270 | 0.51 | .773 |
| Group:AQ.10Z:Condition | 1.49 | .473 | 1.70 | .426 |

## Recency Bias

As previous studies on reinforcement learning in substance use disorder have demonstrated recency bias (i.e., repeating prior response (es) regardless of prior success) (5,6), we conducted an exploratory analysis on the impact of reward history on current choice. We specified three separate variable indicative of reward history from the previous trial; from two trials ago; and for three trials ago. Due to convergence failure, we were not able to include the effect of the group or the interaction effects in the random effect structure.

*Model specification:*

*glmer(stimuliChoice ~ RewardHistoryT1 + RewardHistoryT2 + RewardHistoryT3 + Group + Group:RewardHistoryT1 + Group:RewardHistoryT2 + Group:RewardHistoryT3 + (1 + RewardHistoryT1 + RewardHistoryT2 + RewardHistoryT3 | Participant))*

| **Supplementary Table S22.** Logistic multilevel linear regression model for assessing the relation between reward history and group differences in prosocial learning**.** | | | | |
| --- | --- | --- | --- | --- |
|  | **Lab Study** | | **Online Study** | |
| *Predictor* | *ChiSq* | *p* | *ChiSq* | *p* |
| rewardHistoryT 1 | 56.32 | **<.001** | 93.93 | **<.001** |
| Group | 2.54 | .110 | 0.54 | .459 |
| rewardHistoryT 2 | 33.60 | **<.001** | 44.26 | **<.001** |
| rewardHistoryT 3 | 64.66 | **<.001** | 107.09 | **<.001** |
| rewardHistoryT 1 :Group | 0.64 | .421 | 2.06 | .150 |
| Group:rewardHistoryT 2 | 0.07 | .782 | 0.40 | .526 |
| Group:rewardHistoryT 3 | 0.71 | .399 | 0.01 | .918 |

## Associations between Prosocial Learning and Prosocial Behavior

Here we investigated the role of reduced prosocial behavior in prosocial learning in AUD. Prosocial behavior is reduced in AUD when measured using a dictator game task, or a self-report measure in terms of the altruism subscale included in the prosocial tendencies measure (see Table 1 of the main manuscript for details),

### Prosocial Behavior Using a Dictator Game Task

*Model specification:*

﻿*glmer(HighProbChoice ~ Group + Condition + ProsocBehTaskZ + Group:Condition + ProsocBehTaskZ:Condition + ProsocBehTaskZ:Group + Group:ProsocBehTaskZ:Condition + (1 + ProsocBehTaskZ + Condition + ProsocBehTask:Condition | Participant))*

| **Supplementary Table S23.** Logistic multilevel linear regression model for assessing the relation between prosocial behavior using a dictator game task and group differences in prosocial learning | | | | |
| --- | --- | --- | --- | --- |
|  | **Lab Study** | | **Online Study** | |
| *Predictor* | *ChiSq* | *p* | *ChiSq* | *p* |
| ProsocBehTaskZ | 0.15 | .692 | 1.96 | .161 |
| Group | 0.05 | .822 | 1.34 | .247 |
| Condition | 14.77 | **<.001** | 18.79 | **<.001** |
| ProsocBehTaskZ:Group | 0.09 | .761 | 0.25 | .612 |
| ProsocBehTaskZ:Condition | 3.91 | .140 | 1.00 | .604 |
| Group:Condition | 0.44 | .799 | 2.63 | .268 |
| ProsocBehTaskZ:Group:Condition | 0.07 | .963 | 0.15 | .924 |

### Prosocial Behavior Using a Self-Report Measure

*Model specification:*

﻿*glmer(HighProbChoice ~ Group + Condition + ProsocBehSelfReportZ + Group:Condition + ProsocBehSelfReportZ:Condition + ProsocBehSelfReportZ:Group + Group: ProsocBehSelfReportZ:Condition + (1 + Condition | Participant))*

| **Supplementary Table S24.** Logistic multilevel linear regression model for assessing the relation self-reported prosocial behavior and group differences in prosocial learning | | | | |
| --- | --- | --- | --- | --- |
|  | **Lab Study** | | **Online Study** | |
| *Predictor* | *ChiSq* | *p* | *ChiSq* | *p* |
| ProsocBehSelfReportZ | 0.41 | .521 | 1.10 | .292 |
| Group | 0.17 | .674 | 1.88 | .169 |
| Condition | 16.24 | **<.001** | 21.40 | **<.001** |
| ProsocBehSelfReportZ:Group | 0.02 | .870 | 0.01 | .892 |
| ProsocBehSelfReportZ:Condition | 1.22 | .542 | 0.12 | .940 |
| Group:Condition | 0.56 | .752 | 3.01 | .221 |
| ProsocBehSelfReportZ:Group:Condition | 0.63 | .727 | 3.37 | .185 |

## Computational Modeling of Prosocial Learning

| **Supplementary Table S25.** Model comparison results for the lab study | | | | | | | | | | | |  |
| --- | --- | --- | --- | --- | --- | --- | --- | --- | --- | --- | --- | --- |
|  |  | LLChance | LL | AIC | BIC | αP/* | αS | αN | βP/* | βS | βN |  |
| Model 1 | 1 *α* 1 *β* | 98.85 | 78.67 | 161.34 | 171.26 | 0.30 ^a^ |  |  | 0.69 ^a^ |  |  |  |
| Model 2 | 3 *α* 1 *β* | 98.85 | 77.13 | 162.27 | 182.11 | 0.29 | 0.33 | 0.29 | 0.70 ^a^ |  |  |  |
| Model 3 | 3 *α* 3 *β* | 98.85 | 75.79 | 163.58 | 193.34 | 0.31 | 0.36 | 0.30 | 0.72 | 0.67 | 0.85 |  |
| All reported values are means, and median values supported the same conclusion. LL: log likelihood, AIC: Akaike Information Criterion, BIC: Bayesian Information Criterion, P: Prosocial, S: Self, N: No one. ^a^ parameter is estimated across all three conditions | | | | | | | | | | | |  |

| **Supplementary Table S26.** Model comparison results for the online study | | | | | | | | | | | |  |
| --- | --- | --- | --- | --- | --- | --- | --- | --- | --- | --- | --- | --- |
|  |  | LLChance | LL | AIC | BIC | αP | αS | αN | βP | βS | βN |  |
| Model 1 | 1 *α* 1 *β* | 97.55 | 86.17 | 176.34 | 186.22 | 0.18^a^ |  |  | 0.77 ^a^ |  |  |  |
| Model 2 | 3 *α* 1 *β* | 97.55 | 84.66 | 177.32 | 197.09 | 0.26 | 0.26 | 0.22 | 1.01 ^a^ |  |  |  |
| Model 3 | 3 *α* 3 *β* | 97.55 | 83.58 | 179.17 | 208.82 | 0.24 | 0.25 | 0.25 | 0.97 | 0.97 | 1.23 |  |
| All reported values are means, and median values supported the same conclusion. LL: log likelihood, AIC: Akaike Information Criterion, BIC: Bayesian Information Criterion, P: Prosocial, S: Self, N: No one. ^a^ parameter is estimated across all three conditions | | | | | | | | | | | |  |

| **Supplementary Table S27.** Model selection using BIC weights | | | |
| --- | --- | --- | --- |
|  |  | Lab study | Online study |
| Model 1 | 1 *α* 1 *β* | .956 | .953 |
| Model 2 | 3 *α* 1 *β* | .035 | .046 |
| Model 3 | 3 *α* 3 *β* | .009 | .001 |
| Total |  | 1.00 | 1.00 |
|  | | | |

**Supplementary Figure S15.** Estimated distribution for the alpha and beta parameter for Model 1, for the lab study (A and C) and online study (B and D).


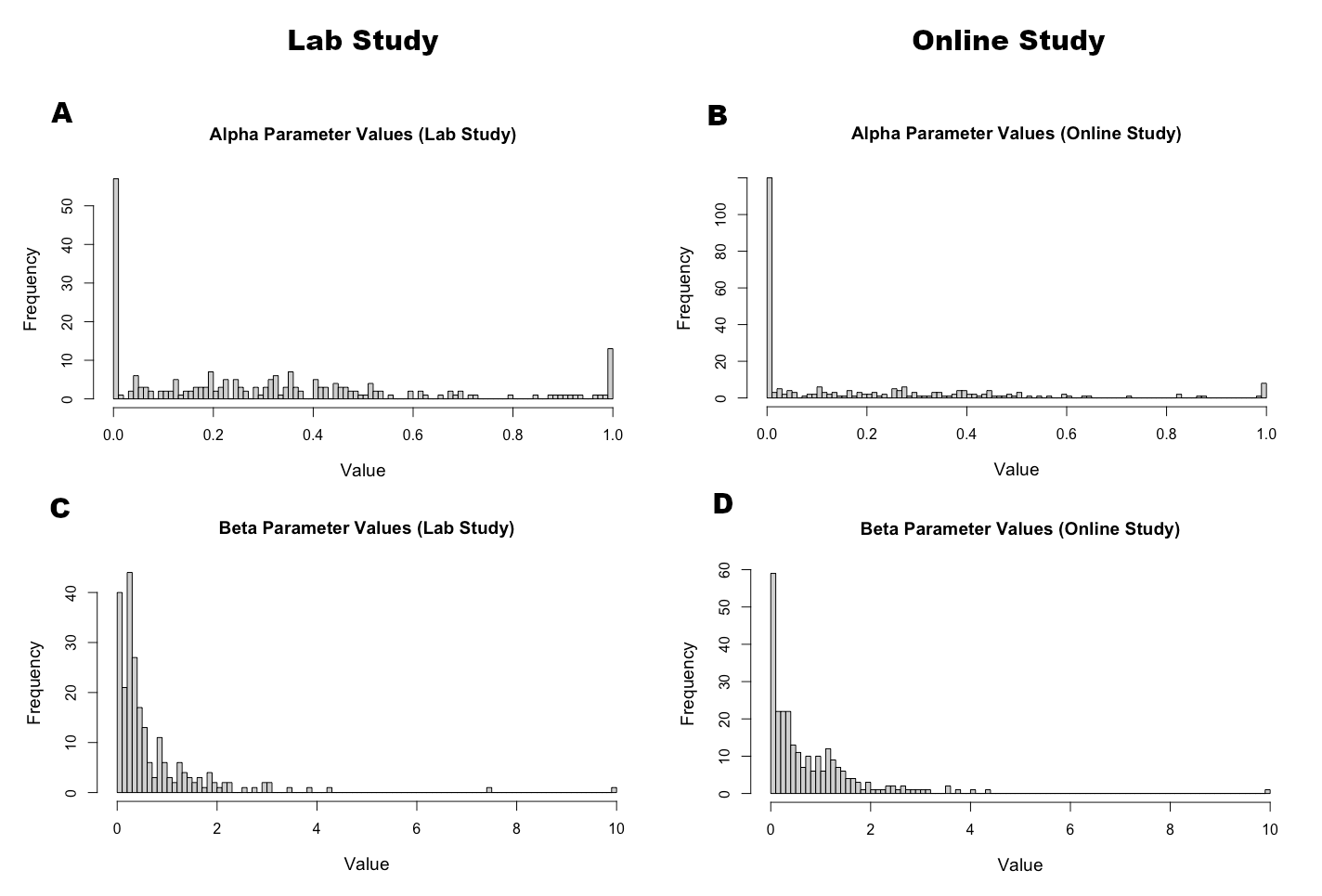


**Supplementary Table S28.** Wilcoxon rank sum test for testing group differences in parameter values for Model 1

|  | **Lab Study** | | | | **Online Study** | |  |  |
| --- | --- | --- | --- | --- | --- | --- | --- | --- |
| *Parameter* | *W* | | *p* |  | *W* | *p* |  |  |
| Alpha~Group | 7353 | | .325 |  | 7173 | .06 |  |  |
| Beta~Group | 6726 | | .823 |  | 7806 | .407 |  |  |
|  | |  | |  | | | | |

# Supplementary References

1. Davis AN, Carlo G, Hardy SA, Olthuis J V, Davis AN, Carlo G, et al. Bidirectional relations between different forms of prosocial behaviors and substance use among female college student athletes. J Soc Psychol. 2017;157(6):645–57.

2. Jangard S, Lindström B, Khemiri L, Pärnamets P, Jayaram-Lindström N, Olsson A. Alcohol Use Disorder Displays Trait-Related Reductions in Prosocial Decision Making. Biol Psychiatry Cogn Neurosci Neuroimaging. 2022;(September):925–34.

3. Carlo G, Randal BA. The Development of a Measure of Prosocial Behaviors for Late Adolescents. Journal of Youth and Adolescence, 31:1 (February 2002), pp 31-44. 2002;31:1(1):31–44.

4. Lindström B, Golkar A, Jangard S, Tobler PN, Olsson A. Social threat learning transfers to decision making in humans. Proc Natl Acad Sci U S A. 2019;116(10).

5. Myers CE, Sheynin J, Balsdon T, Luzardo A, Beck KD, Hogarth L, et al. Probabilistic reward- and punishment-based learning in opioid addiction: Experimental and computational data. Behavioural Brain Research. 2016;296:240–8.

6. Kanen JW, Ersche KD, Fineberg NA, Robbins TW, Cardinal RN. Computational modelling reveals contrasting effects on reinforcement learning and cognitive flexibility in stimulant use disorder and obsessive-compulsive disorder: remediating effects of dopaminergic D2/3 receptor agents. Psychopharmacology (Berl). 2019;236(8):2337–58.
